# The Common Denominators of Parkinson’s Disease Pathogenesis and Methamphetamine Abuse

**DOI:** 10.2174/1570159X21666230907151226

**Published:** 2023-09-07

**Authors:** Bruno Vincent, Mayuri Shukla

**Affiliations:** 1 Institute of Molecular and Cellular Pharmacology, Laboratory of Excellence DistALZ, Université Côte d'Azur, INSERM, CNRS, Sophia-Antipolis, 06560, Valbonne, France;; 2 Chulabhorn Graduate Institute, Chulabhorn Royal Academy, 10210, Bangkok, Thailand

**Keywords:** Methamphetamine, Parkinson’s disease, Parkinsonism, neurodegeneration, neurotoxicity, mitochondrial dysfunction

## Abstract

The pervasiveness and mortality associated with methamphetamine abuse have doubled during the past decade, suggesting a possible worldwide substance use crisis. Epitomizing the pathophysiology and toxicology of methamphetamine abuse proclaims severe signs and symptoms of neurotoxic and neurobehavioral manifestations in both humans and animals. Most importantly, chronic use of this drug enhances the probability of developing neurodegenerative diseases manifolds. Parkinson's disease is one such neurological disorder, which significantly and evidently not only shares a number of toxic pathogenic mechanisms induced by methamphetamine exposure but is also interlinked both structurally and genetically. Methamphetamine-induced neurodegeneration involves altered dopamine homeostasis that promotes the aggregation of α-synuclein protofibrils in the dopaminergic neurons and drives these neurons to make them more vulnerable to degeneration, as recognized in Parkinson’s disease. Moreover, the pathologic mechanisms such as mitochondrial dysfunction, oxidative stress, neuroinflammation and decreased neurogenesis detected in methamphetamine abusers dramatically resemble to what is observed in Parkinson’s disease cases. Therefore, the present review comprehensively cumulates a holistic illustration of various genetic and molecular mechanisms putting across the notion of how methamphetamine administration and intoxication might lead to Parkinson’s disease-like pathology and Parkinsonism.

## 1 INTRODUCTION

Parkinson’s disease (PD) is the second most common neurodegenerative disorder after Alzheimer’s disease (AD), affecting approximately 1% of the population over the age of 60 and 4% over 80 [1]. As such, neurological disorders like PD are a global cause of disability worldwide [2]. The pathological hallmarks of PD are a loss of dopaminergic neurons in the substantia nigra pars compacta (SNpc) and the presence of fibrillary cytoplasmic inclusions, known as Lewy bodies containing α-synuclein, which eventually extend to limbic and neocortical brain regions, consequently decreasing brain dopamine (DA) levels and leading to a severe deterioration of cognitive functions [3]. Alpha-synuclein is genetically and pathologically linked to PD. It is a presynaptic neuronal protein, and in its native form, it is mostly unfolded without a defined tertiary structure in the brain [4]. Still, its soluble aberrant oligomeric conformations mediate the disruption of cellular homeostasis and neuronal death [5]. In PD, α-synuclein becomes prone to aggregate by adapting β-sheet-rich amyloid-like structures, thus forming protofibrils [6].

At the biochemical level, DA accounts significantly for the neuronal communication between the substantia nigra and basal ganglia, which is responsible for the fine-tuning of voluntary movements. PD is characterized by the degeneration of neurons in the SNpc, resulting in substantial loss of DA [7], thus impairing the overall DA metabolism, as observed in PD pathogenesis [8]. Moreover, the role of DA receptors (D1/D2) has been considerably discussed concerning the pathogenicity of PD [9]. Clinical manifestations of PD include prototypal motor symptoms, such as resting tremor, rigidity, bradykinesia, postural instability and slowness of movements, along with cognitive impairment and depression [10]. Apart from abnormal protein aggregation and degradation, multiple pathways are explicitly involved in PD pathogenesis [11], including mitochondrial dysfunction, oxidative and endoplasmic reticulum (ER) stress, neuroinflammation and immune disruption, autophagic dysregulation and apoptosis.

Methamphetamine (meth) is a potent addictive, stimulant drug. The neurobiology of meth is not manacled to be a monoaminergic modulator but extends beyond complex neural systems and biological pathways, where both cerebrovascular and cardiovascular pathologies mitigate the mortality associated with meth abuse [12]. Most importantly, conditions like suicidality, psychosis, depression, and violence contribute to the poor mental health associated with stimulant use, and continuous abuse of such drugs enhances the incidence of HIV and hepatitis C infection, thus upraising the mortality rate [13]. Additionally, patients with prolonged abuse of drugs like meth exhibit a severe phase of meth withdrawal syndrome [14], where clinical analysis reveals drug craving as a salient extrapolate factor in meth-dependent patients with sustained drug abuse [15]. Moreover, a postmodern syndrome called “substance-related exogenous psychosis” (SREP), which has been ratified as a distinct psychotic disorder with psychopathological specificities, has been recently delineated [16]. This notion aids in differentiating this condition from schizophrenia and assists in the differential diagnosis between comorbid conditions, persistent and transient substance-related psychotic states, as well as the preference of treatment interventions with marked specificity as observed in subjects with acute psychiatric presentations post-use of psychoactive substances [17]. Given the broad-spectrum abuse of addictive drugs in urban and rural areas, it becomes essential to focus on preventive strategies concerning the hazardous consequences of unceasing drug intoxication. Novel psychoactive substances (NPS) are a heterogeneous group of substances posing public health threat, thus sabotaging the socio-economic status [18].

As a matter of fact, these substances are analogous to existing controlled drugs and pharmaceutical products mimicking the psychoactive effects of licensed medicines and other controlled substances. The number of drugs that shares the same pharmacodynamic properties is huge and needs to be reported. In many cases, meth is sold as NPS with varied names, and such compounds which share common characteristics with meth have been implicated in cases of psychosis, suicidality [19] and serotonin syndrome [20]. As a matter of concern, NPSs are recurrently sold online as “legal” and “safer” alternatives to internationally controlled drugs [21]. Therefore, web mapping of drug-related issues with preventive strategies must hold immense attention and interest in order to better assess the characteristics and diagnostic challenges of such substances [22, 23].

Strikingly, the genetic propinquity and patterns of neuronal loss and atrophy are quite similar at the gross anatomical level in people who abuse meth and PD patients. Most importantly, besides the fact that chronic meth abuse leads to several neurodegenerative changes in the human brain, meth intoxication resembles symptoms similar to those observed in neurodegenerative disorders like AD [24], PD and early-onset stroke [25]. Even the movement disorders seen in PD resemble what is observed in human meth abusers [26] as well as in animal models like macaque monkeys [27] and rodents [28]. It has been noticed in adult human meth abusers that the prevalence of tremors and abnormal fine hand control was significantly higher than in normal control groups [29], and patients suffering from meth use disorder significantly exhibit extrapyramidal side-effects with evidence for a dose-response effect [30]. Finally, meth abusers present severe defects in hippocampal-related learning and memory performance due to an alteration of the dopaminergic system [31].

Concerning DA homeostasis, a molecular docking analysis revealed that meth and amphetamine act as potential inhibitors of DA receptor for DA uptake [32] and block DA transporter (DAT) and vesicular transport and, therefore, etiologically important in the cytosolic DA mediation of neurodegeneration in PD/Parkinsonism [33]. Additionally, both DA receptors (D1 and D2) are involved in meth-induced neurotoxic mechanisms [34]. Finally, a recent investigation carried out in mice and neuronal cells has revealed that meth induces a loss of dopaminergic neurons and activation of autophagy mediated by D1 receptors and involving the AMPK/FOXO3A signaling pathway (He *et al.*, 2022).

Among other related receptors, the sigma receptor has the ability to act on different monoaminergic pathways for which it is considered to affect both motor and non-motor PD symptoms [35] and interestingly sigma receptor has a key role in meth-induced deregulation of DA release and DA-related behaviours [36]. Comprehensive descriptions of meth-induced dopaminergic neurodegeneration and its relevance to PD from pertinent findings from human and animal studies have been relatively documented [37].

Pertaining to α-synuclein pathology, it has been shown that meth significantly increased the expression of α-synuclein in the substantia nigra of the rats [38]. Additionally, it has been evidenced that chronic meth exposure increased α-synuclein levels in the stratum oriens, pyramidal layer, stratum radiatum and stratum moleculare of hippocampal CA1, CA2 and CA3, polymorph layer of the hippocampal dentate gyrus, and substantia nigra of mice [39]. Moreover, chronic meth abusers demonstrate significant α-synuclein overexpression and aggregation in dopaminergic neurons in the substantia nigra, similar to the α-synuclein cytotoxicity in PD cases [40], which is remarkably associated with neuronal loss and motor dysfunction in patients with PD.

Considering the above-mentioned imperative explorations, the present review exemplifies a comprehensive illustration of meth-induced pathogenic mechanisms similar to PD pathology, thus establishing a link for further investigations and directed therapeutic implementations.

## 2 GENETIC LINKAGE BETWEEN DRUG ADDICTION AND PD

Effective development of gene therapies requires not only the identification of specific molecular and anatomic targets but also specific brain regions or networks to modulate with genetic intervention [41]. In this context, it is of utmost interest to note that polymorphism in the synuclein alpha (SNCA) gene is also associated with meth psychosis [42]. Thus, given the fact that amyloid beta (Aβ), tau and α-synuclein interact, modulate and enhance each other’s [43], an additional synergistic contribution of meth could further accelerate cognitive dysfunction during the time-course of AD, PD, and dementia with Lewy bodies (DLB). Now regarding specific genetic variations linking drug abuse and PD, although PD has been described as a prototypical sporadic disease, advancement in molecular genetic studies has revealed this neurodegenerative disorder as a genetic disorder [44], with the traditional model of PD being SNCA-centric, although the microtubule-associated protein tau (MAPT) gene locus has come out to be also a risk factor for PD [45].

Interestingly, abusing meth increases threefold the risk of developing PD by inducing conformational changes in α-synuclein structure [46] and augments α-synuclein protein levels in the hippocampus of adolescent mice [47]. From a genetic point of view, when considering that single nucleotide polymorphisms in the a-synuclein gene SNCA are strongly associated with PD risk [48, 49], it is of utmost interest to underline here that, as mentioned previously, polymorphism in the SNCA gene is also associated with meth psychosis [42]. Moreover, meth exposure causes persistent demethylation within the SNCA promoter, corresponding to the stoichiometric steady augmentation of α-synuclein protein levels within the striatal neurons [50].

In addition, the Val/Met single nucleotide polymorphism at codon 66 of the brain-derived neurotrophic factor (BDNF) gene has been associated with a higher risk for meth abuse [51], while it has also been linked to cognitive decline in PD [52]. Interestingly, the link between BDNF, PD and drug abuse was further supported by the fact that serum levels of BDNF, which regulates not only synaptic plasticity but also fulfills diverse roles in addiction-related behaviours [53], are associated with the cognitive state in PD patients with mild cognitive impairment (MCI) [54]. Moreover, a recent meta-analysis of human meth users revealed a significant correlation between meth users and BDNF Val66Met polymorphism [55]. Ultimately, in addition to BDNF and extending beyond purely genetic factors, modifications in the levels of other proteins have been observed in both PD and meth-related conditions. Hence, levels of regulator of G-protein signalling 2 (RGS9) are abnormally high in PD [56], while its involvement in schizophrenia and the development of meth-induced psychosis has been clearly established [57].

Importantly, among the known genes associated with PD (SNCA, Parkinsonism Associated Deglycase (PARK)7, Leucine-rich repeat kinase 2 (LRRK2), PARK2, or PTEN-induced kinase 1 (PINK1), most of them require thorough investigations to establish a link between them and meth abuse, however, some relevant links cannot be ruled out. Mutations in the PARK7 gene are known to cause rare forms of early-onset PD, and interestingly PARK7 interacts with p47phox to direct nicotinamide adenine dinucleotide phosphate hydrogen (NADPH) oxidase-dependent reactive oxygen species (ROS) production [58]. Therefore, it would be challenging to speculate whether or not meth’s interaction with p47phox involves PARK7. Yet another gene is the glucocerebrosidase (GBA1), encoding the lysosomal enzyme glucocerebrosidase, which heterozygous mutations have been linked to PD development and related synucleinopathies [59]. Moreover, along with SNCA and apolipoprotein (APOE), epigenetic modifications and genetic variations in GBA1 are involved in DLB [60]. Meth dose-dependently not only decreased the expressions of the α-synuclein-specific degradative enzyme glucocerebrosidase but simultaneously also reduced the levels of its regulator lysosomal integral membrane protein type-2 in pheochromocytoma (PC12) cells [61], indicating a relevant annexation in between.

Overall, the association of addiction-related variants with enhanced risk for developing degenerative brain disorders [62, 63], decline in cognitive performances [64] and differences in brain structure [64, 65] are key factors in understanding the pathobiological link between them (Table **1**).

## 3 METHAMPHETAMINE-INDUCED STRUCTURAL ALTERATIONS IN THE BRAIN WITH REFERENCE TO PD

Meth-induced neurotoxicity is reflected by the structural abnormalities in the brain exposed to its chronic use. One striking resemblance between meth pathology and PD concerns the superposition or at least the interconnectivity of the affected brain areas. Firstly, the nucleus accumbens, a key structure of the reward centre and addiction, is part of the striatum and displays close interconnections with both limbic structures (hippocampus and amygdala) and the prefrontal cortex (PFC) so that it may integrate information involved in learning and executive function that are affected in PD. Even prenatal meth exposure leads to structural and functional alterations of striatal, frontal, parietal, and limbic regions, as affirmed by brain imaging studies [66].

Meth causes severe energetic metabolism impairment in the amygdala, PFC, hippocampus and striatum of rats that is accompanied by a significant behavioural sensitization [67, 68]. Precisely, meth exposure in rats decreased striatal volume and dendritic length associated with enhanced astrogliosis and deregulated miRNAs in the striatum with abatement of motor coordination [69]. Moreover, self-administration of meth in rats results in a loss of corticostriatal plasticity and impaired motor learning [70].

**Fig. (1):**
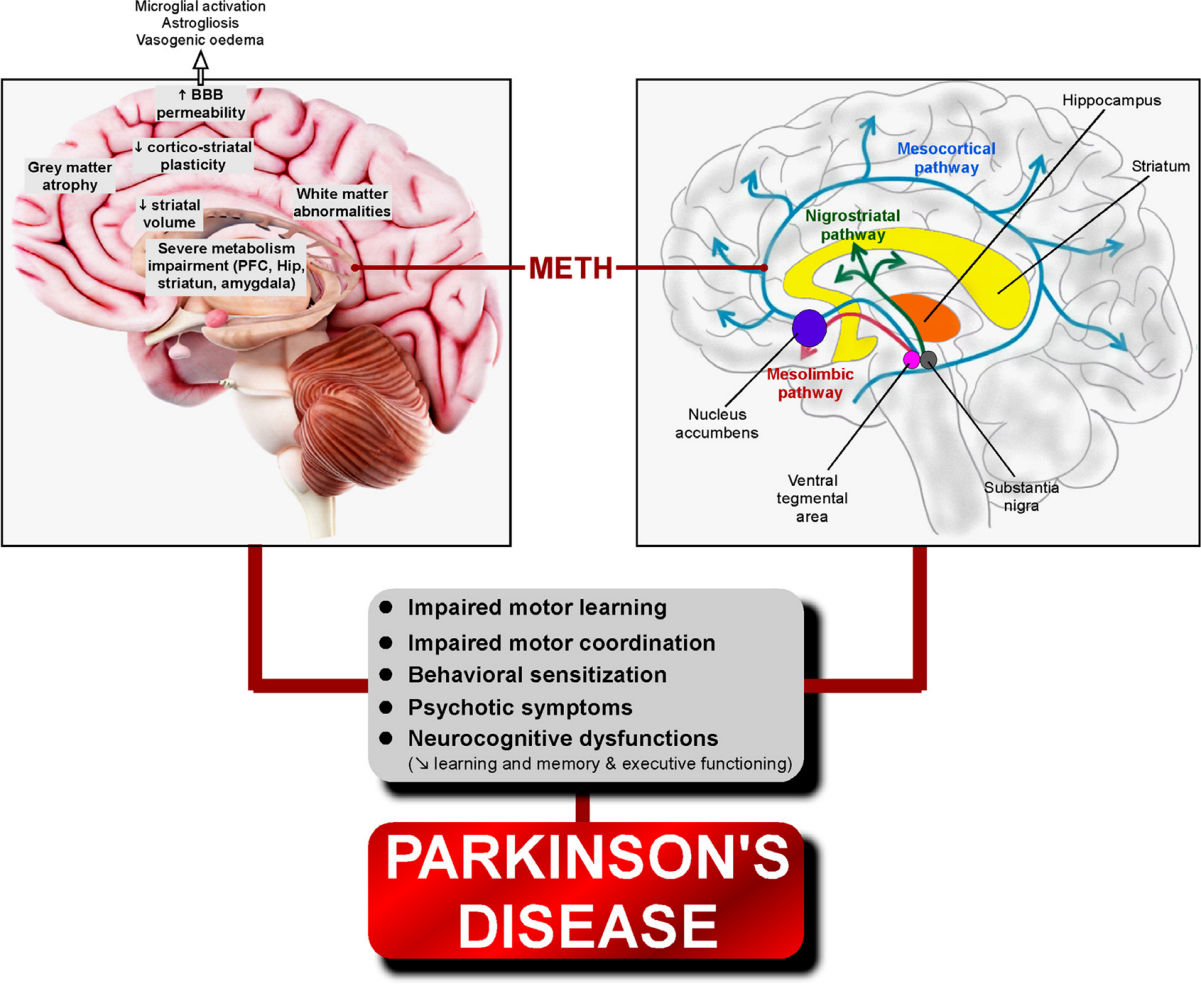
Schematic outline of the similarities in the brain circuits and regions affected, the damage caused and the resulting effects observed in methamphetamine-induced neurotoxicity and PD pathogenesis. Firstly, meth profoundly affects the BBB permeability, thereby resulting in enhanced microglial activation and astrogliosis. Secondly, meth leads to significant abnormalities in both grey and white matter and cause metabolic impairments in different brain areas as observed in PD pathology. Thirdly, meth, by modifying the nigrostriatal, mesocortical and mesolimbic dopaminergic pathways, leads to motor impairments (locomotor activity and motor coordination) resulting from a loss of neurons in the substantia nigra. Finally, meth, by affecting these pathways, is responsible for cognitive dysfunctions (learning and memory impairments) and neuropsychiatric disturbances like depression, psychosis, along with behavioral changes, which are characteristic features of PD pathogenesis.

Importantly, advanced diagnostic techniques such as diffusion kurtosis imaging scanning using the Bruker Avance 9.4 Tesla magnetic resonance imaging (MRI) system tend to reveal microstructural brain changes during neurodegeneration. Thus, a comparative voxel-based analysis had previously shown gray matter volume reductions in the brain of PD patients associated with other structural abnormalities [71, 72]. More recently, whole-brain voxel-wise and region-of-interest-wise causal structural covariance network approaches allowed to show a progressive augmentation of gray matter atrophy from the basal ganglia to the angular gyrus, temporal areas, eventually spreading through the subcortical-cortical networks in accordance with the progression of pathology observed in PD patients [73]. It is assumed that white matter impairment in PD might be a sensitive sign preceding the neuronal loss in associated grey matter regions [74]. In this context, computational brain mapping techniques demonstrated decrements in gray matter volume in the paralimbic, limbic and cingulate cortices of meth abusers [75]. This is consistent with the fact that meth abusers display significant abnormalities in the grey and white matter of their brains [76-79]. Additionally, recently developed brain imaging techniques have evidenced grey matter structural and volumetric alterations in meth users [80] and PD patients [81], thus establishing a similarity in the structural changes occurring in PD and meth abuse and suggesting a possible causal link between the two (Fig. **1**). This hypothesis gains further support from the discovery that employing a comparable technique has revealed microstructural pathological processes in both grey and white matter have been detected in a meth-induced mouse model of PD [82].

MRI investigations in chronic meth-treated rat models demonstrated enlarged striatal volumes and increases in [3H]PK 11195 binding in the frontal cortical areas, the rhinal cortices, the striatum, the nucleus accumbens and the cerebellar nuclei [83]. Recently, voxel-based morphometry in conjunction with statistical parametric mapping on structural magnetic resonance images demonstrated higher Barratt Impulsiveness Scale (BIS-11) impulsivity scores and a lower grey matter volume in the bilateral superior frontal cortex in individuals with severe meth use disorder, indicating higher impulsivity [84].

Furthermore, circular RNAs are known to have an important role in neurodegenerative disorders like PD [85]. These are stable noncoding RNAs that accumulate with aging and are involved in the regulation of neuronal functions. Meth profoundly changes the profiling of circular RNA expression in the cerebellum of rats with significant alterations in motor coordination and muscle activity [28]. Cytochrome P450 2D6 (CYP2D6) polymorphisms have been linked to PD susceptibility [86]. Being centric on meth metabolism, any genotypic variations in CYP2D6 modulates meth effects on brain structure, function, and cognition [87].

## 4 METHAMPHETAMINE-INDUCED MOLECULAR PATHWAYS IMPACTING PARKINSON’S DISEASE PATHOGENESIS

This section discusses how meth abuse might be a triggering event in developing PD and Parkinsonism. Indeed, evidence-based studies in animal models and clinical and population assessments have revealed signs of prodromal and emerging PD among meth users [88].

Meth can trigger Parkinsonism symptoms at high doses or following long-term exposure due to its capability to cause dopaminergic neurodegeneration, similar to what is observed in PD [89] as observed in studies carried out in rodents and primates [90, 91]. High-dose meth treatment in mice results in a loss of DA cells in the SNpc [92], an area mainly affected in PD, which includes the substantia nigra, the basal ganglia and the cerebral cortex. Compared to other amphetamine derivatives, meth depletes DA faster and causes a long-lasting impact on DA levels. Consequently, it induces a long-lasting degeneration of dopaminergic cell bodies in the SNpc, along with the destruction of dopaminergic terminals in the striatum [93].

DA receptors (D1-D5) are involved in the regulation of numerous physiological functions in the brain and periphery, and their signalling mechanisms and mode of action play a significant role in neurodegenerative pathomechanisms [94]. These receptors mediate a diversity of functions: behaviour and cognition, voluntary movement, motivation, punishment and reward, attention, working memory and learning [34] and are involved in the genesis and pathophysiology of PD [9]. A recent systematic review and meta-analysis of the positron emission tomography (PET) and single-photon emission computed tomography study investigated DA receptors in PD patients, which indicated that the observed compensatory receptor changes in the study were an outcome of the loss of DA nerve terminals and striatal neuropil with subsequent neurodegeneration [95]. Interestingly, these receptors are the mediators of meth-induced neurotoxicity [34], promoting ER stress and mitochondrial dysfunctions in the striatum of rodents [96], which is reminiscent of the pathological mechanisms observed in PD.

### 4.1 PD-promoting Deleterious Meth-dependent Pathways

#### 4.1.1 Gene Expression, Epigenetic and miRNA-mediated modifIcations

**Fig. (2):**
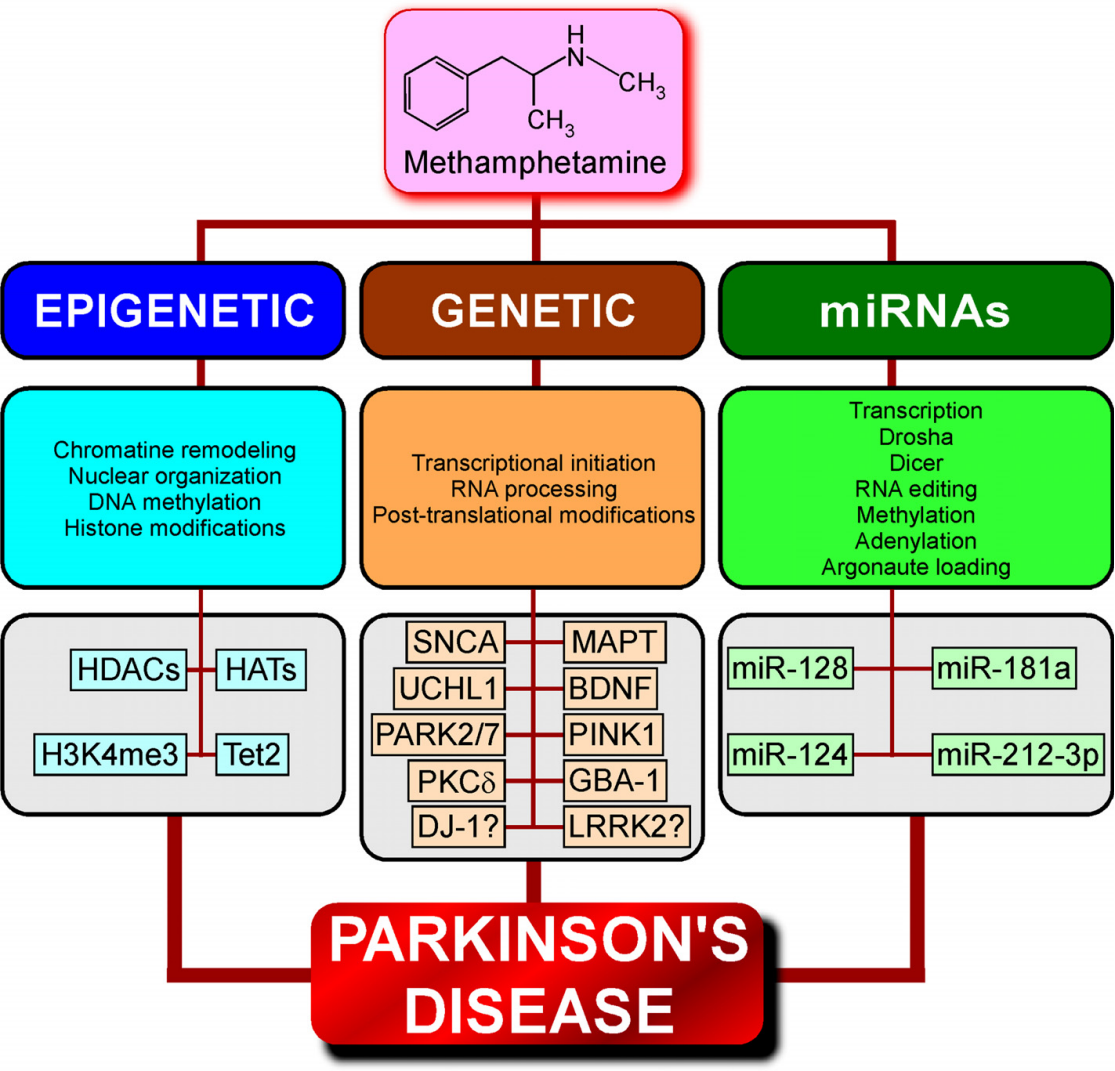
Schematic representation of methamphetamine-induced modulations in epigenetic processes and gene and miRNA expressions thought to induce Parkinson's disease. Meth, *via* epigenetic modulation affects the functional complexity of DNA by altering chromatin structure, nuclear organization, methylation, and histone modifications. Furthermore, meth alters, through transcriptional and post- transcriptional modifications, the expression of certain genes involved in PD. Finally, meth alters the biogenesis profiles of some miRNAs, thereby possibly playing a crucial role in inducing PD-like pathology.

Genetic and epigenetic mechanisms, such as DNA methylation, histone modifications (acetylation and methylation) and small RNA-mediated mechanisms, play important roles in PD pathogenesis by regulating the expression of genes relevant to PD [97, 98], where redundant exposure to drugs like meth modulates both DNA methylation status and post-translational histone modifications in several regions of the brain [99, 100]. Noteworthy, meth abuse is associated with extensive gene expression changes in various brain regions [101-103] and has been shown to induce major epigenetic modifications [103-105], which may aid in understanding the link between meth-induced modifications and the aetiology, pathophysiology and progression of PD pathology (Fig. **2**).

The epigenetic and transcriptional upregulation of Tet methylcytosine dioxygenase 2 (TET2), a master regulator of cytosine modification status, observed in PD patients is considered a causative factor in widespread epigenetic dysregulation of PD neurons [106]. Interestingly, it has been demonstrated that meth induces DNA hydroxymethylation of certain genes in the nucleus accumbens in a TET1- and TET3- dependent manner, thus providing molecular evidence for epigenetic regulation of meth-induced alterations in gene expression [107]. Whether a connection exists between meth and TET2 will certainly deserve further investigation to establish an additional common denominator between meth abuse and PD pathogenesis.

Deregulation of histone deacetylases (HDACs) is considered a potential contributor to aberrant transcriptional profiles that leads to alterations in cognitive functions, and upregulation of HDAC2 has been observed in PD [108]. It is to highlight that meth has been shown to differentially regulate HDAC superfamily promoters acetylation [109, 110], with meth administration increasing the expression of HDAC2 protein in the rat nucleus accumbens [103]. It is, therefore, apprehensible that the plausible meth-induced imbalances in the actions of HATs/HDACs could cause deregulation of transcription and disturbance in the neuronal homeostasis in disorders such as PD.

The genes that have been extensively studied regarding PD pathology are SNCA, parkin, PINK1, Protein deglycase *DJ-1* (DJ1) and LRRK2 since the abnormal aggregation and/or mutation of these proteins/genes have been observed in PD [111]. As an example, a loss of function and/or mutations in parkin is associated with an autosomal juvenile form of PD as a parkin gene defect is involved in the selective degeneration of dopaminergic neurons. In this context, it is interesting to note that meth decreases the expression levels of parkin and its substrate Pael-receptor in the striatum of rat brain [112], which implies that modulation of parkin and Pael-R genes by meth would potentially transmogrify the pathophysiology of the protein and favour the development of PD.

Other putative meth-regulated PD-associated factors are LRRK2 and SNCA, where epigenetic deregulation of α-synuclein plays a crucial role in PD pathology. Recently, it has been demonstrated that H3K4me3 (an epigenetic modification to the DNA packaging protein Histone H3), which regulates α-synuclein, was significantly elevated at the SNCA promoter of the substantia nigra of PD patients observed during both punch biopsy and in NeuN (neuronal nuclear protein)-positive neuronal nuclei samples [113]. In this regard, it has been shown that the transcriptional response of midbrain dopaminergic neurons following meth injection is characterized by an enhanced expression of genes with promoters dyadically marked by H3K4me3/ H3K27me3 [114]. The LRRK2 gene is responsible for the most common familial form of PD with autosomal dominant inheritance [111] and regulates α-synuclein neuropathology in PD [115, 116]. Although the SNCA gene is associated with meth psychoses [42], so far, there is no direct evidence of meth-induced regulation of LRRK2. However, considering that increasing the level of let-7 attenuates the pathogenic effects of LRRK2 [117], the fact that the micro-RNA miR-let-7e decreases in the plasma of meth abusers strongly implies that meth could indirectly modulate LRRK2 physiology [118].

Besides the “classical” PD-associated genes/proteins mentioned above, several other factors shown to be linked to the pathology are under the regulation of meth. Indeed, according to the Kyoto Encyclopedia of Genes and Genomes (KEGG) pathway database, some genes related to DA re-uptake (Comt, Slc family) or some DA-regulated downstream signals such as protein kinase B (Akt), glycogen synthase kinase (GSK3) α/β, protein phosphatase 2A (PP2A), and phospholipase C (PLC) could possibly be modulated by meth [119]. As an example, acute meth exposure increases the rat cortical expression of the ubiquitin carboxy-terminal hydrolase L1 (UCHL1) [120], which has been identified as a PD-associated gene [121]. Moreover, histone acetyltransferase p300 (HATp300) enhances the aggregation of misfolded proteins in cell models and Lewy bodies of PD patients containing α-synuclein [122], and meth administration significantly increases the protein expression of histone acetyltransferase in the rat nucleus accumbens [103]. The neurotrophic factor BDNF boosts neuroregeneration and furnishes neuroprotection, and investigations in animal models of PD have manifested improvements in dopaminergic neurotransmission and motor performance with an overall enhancement in the survival of dopaminergic neurons [123]. Concerning the fact that meth dependence both in humans and in animal models increases BDNF methylation [124], the alterations in its expression and function would significantly affect the neuroprotective properties of this neurotrophic factor.

The growing evidence of miRNAs' involvement in regulating various disease processes, including PD [125] and drug addiction, makes them potential drug targets. The fact that some miRNAs are regulated by meth [126] provides molecular grounds for revealing the mechanisms underlying meth addiction and neurotoxicity. Meth is known to be involved in the regulation of the levels of Dicer1 and Argonaute2 proteins, that are both account for miRNA silencing complexes [127]. Interestingly, altered levels of Dicer1 [128] and reduced levels of Argonaute 2 [129] in PD patients might be indicative of how meth could play a crucial role in the pathoetiology of PD by modulating the biogenesis profile of some significant miRNAs. Moreover, analysis of miRNAs profile using the Illumina HiSeq™ 2500 sequencing system showed some substantial alterations in meth-addicted rats in the nucleus accumbens [130], a region whose atrophy has been previously suggested to be involved in both motor and neuropsychiatric symptoms of PD [131].

For instance, miR-128 stands as a potential target for PD therapeutics since its expression affects apoptotic mechanisms in DA neurons along with the expression of excitatory amino acid transporter 4 (EAAT4) [132], which are high-affinity glutamate transporters. Dysfunction of EAATs and alterations in their expression have been revealed in PD animal models [133]. Meth-induced behavioural sensitization counts on long-term neuroplasticity in the mesolimbic DA system. Recently, it has been suggested that miR-128 is involved in regulating meth sensitization through controlling neuroplasticity [134].

Interestingly, miR-181a negatively controls parkin [135], which is a key factor in PD pathomechanisms [111] and, as a consequence, directly impairs the expression of the glutamate ionotropic receptor AMPA type subunit 2 (GRIA2) [136]. Interestingly, chronic meth use reduces the expression of miR-181a [118]. Altogether, these findings support the probability that meth, through its action on the miR-181a/ parkin/GRIA2 signaling axis, could participate in the development of PD. Moreover, a bioinformatics analysis recently revealed that miR-181a may indirectly be responsible for meth addiction by regulating ER-associated protein degradation [137].

A study has also established that miR-124 is altered in a 1-methyl-4-phenyl-1,2,3,6-tetrahydropyridine (MPTP)-treated mouse model of PD [138], supporting the fact that miR124 is a factor that could putatively favour PD pathology. Interestingly, miR-124 was also associated with meth addiction [139], thereby suggesting a role for this micro-RNA in meth-induced PD. Recently, it has been shown that miR-212-3p is downregulated in PD [140] and in the nucleus accumbens of meth-treated mice [119], which demonstrates yet another additional way through which meth could promote PD. A summary of the data in this section is presented in Table **2**, and additional evidence-based study in this particular area of investigation would certainly make us better understand the complex gene regulatory network involved in meth addiction and PD-like pathology.

#### 4.1.2 Blood-brain Barrier Integrity Disruption

The blood brain barrier (BBB) is a tightly regulated interface in the central nervous system (CNS) that regulates the exchange of molecules in and out from the brain, thus maintaining CNS homeostasis; there is growing evidence that during aging and in neurodegenerative disorders such as PD, there is an alteration of structure and function of the BBB including a loss of efficiency of tight junctions and efflux transporters [141]. This phenomenon consequently elicits peripheral immune response, vascular density changes and, most importantly, altered drug efficacy [142] with phenotypical changes in endothelial cells and astrocytes with profound reactive gliosis damaging neuronal survival [143]. Specifically regarding PD patients, a significant increase in BBB leakage [144] and permeability of the BBB occurs in post-commissural putamen [145]. Consequently, the altered transport of *α-synuclein,* a presynaptic neuronal protein that is linked genetically and neuropathologically to PD, *via* the BBB, might result in its aggregation leading to PD pathoetiology [146] presenting, due to this particular feature, a target of choice for therapeutic strategies aimed at treating PD [147]. In this context, it has been shown that dynamic changes in vessel morphology and compromised BBB integrity occurs in an α-synuclein-overexpressing mouse model, which exhibits the characteristic pathological features of PD [148], thereby further affirming that such microvascular alterations exacerbate neurodegeneration.

Meth itself can cause structural and functional disruption of the BBB with the methyl group making the molecule more lipophilic and thus facilitating transport across the BBB [149]. Indeed, meth induces an increase of BBB permeability in the rat hippocampus and striatum, thereby triggering structural alteration of blood vessels and decreasing the levels of intercellular junction protein such as claudin-5, occludin and vascular endothelial cadherin, along with microglial activation, astrogliosis and increased pro-inflammatory mediators [150]. As a probable consequence, meth administration causes significant structural and functional changes in the brain, as evidenced in both human meth abusers [75] and rats [83]. In complicated chronic conditions, meth-induced leakage of BBB, which is considered temperature-dependent, is followed by vasogenic oedema and ultimately leads to death [151].

Notably, inhibition of the pentose-phosphate pathway (PPP), which ensures the proper oxidative status of neurons, causes selective dopaminergic cell death leading to motor deficits resembling Parkinsonism and dysregulation of glucose metabolism, which is an early event in sporadic PD [152]. Thus, as meth alters brain glucose metabolism in various brain regions [153], one could hypothesize that meth, through its deleterious action on glucose homeostasis, would promote the development of PD *via* the alteration of BBB integrity.

Some recent studies have shown that meth causes cerebrovascular alterations [154] and induces endothelial cell death, where neuropeptide Y plays an important role in meth-mediated neuronal and glial toxicity. Using a human brain microvascular endothelial cell line (hCMEC/D3), it has been shown that meth exposure altered the expression of neuropeptide Y2 receptors [155], which are apparently involved in providing neuroprotection as observed in animal models of PD [156]. Meth administration also significantly enhances ROS generation, induces the formation of robust stress fibers causing reorganization of the cytoskeletal and alters the cellular localization of the tight (ZO-1) and VE-cadherin in the primary human brain microvascular endothelial cells [157]. Moreover, it triggers an excessive increase in matrix metalloproteinase-9 (MMP-9) enzyme, intercellular adhesion molecule 1 (ICAM-1) and vascular cell adhesion molecule 1 (VCAM-1) along with an increase in NAD(P)H oxidase 2 (NOX2) in the hippocampal and prefrontal cortical tissues of rats [158]. Finally, meth induces the overexpression of RhoA, Rho-associated protein kinase (ROCK), myosin light chain (MLC), cofilin, phosphorylation (p)-MLC, p-cofilin and MMP-9 in rats, thereby suggesting that meth might increase BBB permeability also through the activation of the RhoA/ROCK pathway [159].

#### 4.1.3 Endoplasmic Reticulum Stress

ER, stress enables cells to overcome the abnormal accumulation of unfolded/misfolded proteins and is a complex process that involves the activation of three major signalling pathways (Activating transcription factor 6 (ATF6), Inositol-requiring enzyme-1α (IRE1α) and Protein kinase RNA-like endoplasmic reticulum kinase (PERK)) [160]. PD etiology and pathology are intimately linked to ER stress and unfolded protein response (UPR) activation [161], as illustrated by the signs of ER stress observed in post-mortem tissue from sporadic human PD cases and in most animal models of the disease [162]. At the molecular level, ATF6, X-Box Binding Protein 1 (XBP1), and C/EBP homologous protein (CHOP) have a functional role in controlling dopaminergic neuron survival in neurotoxin-based models of PD *in vivo* [163]. The Sigma-1 receptor (Sig-1R), which is a chaperone protein located at the mitochondrion-associated ER membrane and associated with calcium signalling between the two organelles, has been considered a potential target for PD as it regulates mechanisms of both cellular defense and damage [35]. In addition, apart from other pathogenic mechanisms, α-synuclein oligomers exert neurotoxicity and promote neurodegeneration *via* ER stress and proteostasis dysregulation [164].

The growing assumption that meth could have an impact on PD development through ER stress enhancement came from various observations. Firstly, meth is able to mediate ER stress *via* all ATF6, IRE1α and PERK signalling pathways [165]. Secondly, there is a positive correlation between ER stress and meth-induced neurotoxicity [166]. Thirdly, meth exposure leads to ER stress in dopaminergic cells [167]. Fourthly, the PD-associated Sig-1R is involved in meth-induced microglial apoptosis and death and blocking this receptor significantly inhibits the generation of ROS and the activation of mitogen-activated protein kinase (MAPK) and Akt pathways [168].

Meth has been shown to induce ER stress through the overexpression of ER stress-related genes, including CHOP and spliced XBP1 [169] and, by augmenting DA levels, triggers ER stress and oxidative stress signalling pathways even in a fetal brain exposed to meth, thus impacting learning and cognitive abilities with significant neurobehavioral deficits [170] similar to some of the PD pathological manifestations. A post-mortem human striatum investigation revealed an upregulation of CHOP, Tribbles homolog 3(Trib3), Nuclear protein 1 (NUPR1) and Beclin 1 in long-term meth abusers when compared with their respective controls along with effective neuronal cell death [171]. This recent study establishes that meth-induced ER stress causes overexpression of NUPR1, which is associated with the upregulation of the pro-apoptotic transcription factor CHOP. Moreover, it has been hypothesized that meth-induced ER stress might play a pivotal role in the upheaval of drug memory where its excessive consumption inhibits drug-evoked synaptic plasticity involving cyclin-dependent kinase 5 (Cdk5) activation and decrement of Ca^2+^/calmodulin-dependent protein kinase II (CAMKII) as demonstrated in mice [172]. Noteworthy, even sub-acute meth ingestions in mice inhibit long-term memory acquisition and synaptic plasticity *via* ER stress [173].

Concerning the meth-induced ER stress-mediated mechanisms of cellular toxicity and cell death, the role of Gasdermin-E (GSDME), the precursor of a pore-forming protein that converts non-inflammatory apoptosis to pyroptosis, a detectable feature observed in the biological fluids of PD patients [174], has been evidenced [175]. Indeed, meth-induced neuronal cell death, specifically by pyroptosis, occurs *via* ER stress-mediated by GSDME in hippocampal neuronal cells [176]. Finally, ER stress mediates meth-induced BBB damage [177], a phenomenon reminiscent of what occurs in PD, as discussed previously [145].

#### 4.1.4 Mitochondrial Dysfunction and Oxidative Stress

Mitochondria are intimately involved in various key cellular processes, such as the regulation of calcium homeostasis, stress response and cell death pathways and therefore represent a highly promising target for the development of PD biomarkers [178]. Mitochondria maintain cellular homeostasis by producing adenosine triphosphate (ATP) and regulating ROS, which is essential for neuronal function [179]. Therefore, neurons attempt to maintain mitochondrial levels in PD to facilitate neural transmission as a compensatory mechanism.

Substantial findings have revealed that genes like Parkin, PINK1, DJ-1, SNCA and LRRK2 are involved in mitochondrial pathways, suggesting a critical role for mitochondrial dysfunction and associated oxidative stress in idiopathic and monogenic PD [180]. To be more precise, α-synuclein interacts with the translocase of the outer membrane (TOM) complex and affects the mitochondrial import. Mutations in LRRK2 affect the tethering of ER-mitochondria and calcium homeostasis; DJ-1 is associated with increased ROS production, while PINK1 and parkin cause defective mitophagy [181]. Moreover, alterations in DNA polymerase subunit gamma or mitochondrial polymerase gamma (POLG) (essential for mitochondrial DNA replication and repair) manifest signs of Parkinsonism [182]. Although meth interactions with some of the genes mentioned above have been established, more thorough investigations are required for further validation.

Like meth, 1-methyl-4-phenyl-1,2,3,6-tetrahydropyridine (MPTP) is a neurotoxin that also crosses the BBB and causes Parkinsonian syndrome by inhibiting mitochondrial respiration [183, 184]. The acute loss of parkin or PINK1 function causes dynamin-related protein 1 (DRP1)-dependent mitochondrial fragmentation along with a decrease in the mitochondrial membrane potential and ATP production [185, 186]. In the transgenic mouse model of PD, alterations in the functioning of DRP1 are associated with α-synuclein pathology [187], while meth-induced modifications of parkin were found to significantly decrease DRP1 levels [188].

Mitochondrial dysfunction is a key determinant of dopaminergic neuronal susceptibility and is a feature of both familial and sporadic PD [189, 190]. A plethora of mutations in mitochondrial DNA, nuclear DNA gene mutations, alterations in mitochondrial dynamics, alterations in trafficking/ transport and mitochondrial movement, abnormal size and morphology, impairment of transcription and the presence of mutated proteins associated with mitochondria are implicated in PD [191]. Meth inhibits mitochondrial function, increasing the free radical burden and decreases neuronal energy supplies [192] and further inducing mitochondrial fragmentation, apoptosis, and inhibiting cell proliferation in rat hippocampal neural progenitor cells [193]. Both *in vivo* and *in vitro* studies have suggested that mitochondrial dysfunction is crucial in meth-induced dopaminergic toxicity as it further enhances pro-apoptotic mechanisms, oxidative stress and neuroinflammation and where PKCδ has been considered as a prompt mediator [194]. Interestingly, PKCδ-induced neuroinflammation has been documented in PD and other synucleinopathies [195].

In PD, degeneration of the dopaminergic nigrostriatal pathway leads to enhanced transmission of NR2B subunit-containing N-methyl-D-aspartate (NMDA) receptors [196]. Similarly, meth increases the expression of NMDA receptor subunit 2B (NR2B) and the level of glutamate in the ventral tegmental area (VTA) and nucleus accumbens (NAc) of mice [197]. Interestingly, glutamatergic hyperactivity has been extensively observed in nigrostriatal pathways in PD [198].

It has been established that mitochondrial and lysosomal dysfunctions are associated with α-synuclein pathogenicity [199]. In PD, the generation of protein aggregates may disrupt the mitochondrial membrane potential and induce abnormal calcium influx, impair the respiratory enzyme activities, reduce ATP generation, and increase levels of ROS. Also, the abnormal release of cytochrome C from damaged mitochondria can trigger the activation of the apoptotic signalling cascades and the release of caspases, resulting in neuronal cell death. Altogether, DA, iron, calcium, mitochondria and neuroinflammation contribute to the overwhelmed oxidative stress and neurodegeneration in PD [180, 200]. In this context, meth causes a toxic malady that is characterized by altered carbohydrate metabolism, dysregulation of calcium and iron homeostasis, increased oxidative stress and disruption of mitochondrial functions [201], which involves toll-like receptors and nuclear factor kappa light chain enhancer of activated B cells (NFκB) as some of the important underlying signalling mechanisms [202]. Moreover, meth exposure significantly decreases the activity of nuclear factor erythroid 2-related factor 2 (Nrf2) and the expression of its downstream proteins [203], Nrf2 being a ubiquitous master transcription factor that upregulates antioxidant response elements-mediated expression of antioxidant enzymes. A very recent investigation has revealed the beneficial effects of Nrf2 expression in inhibiting the progression of PD in 6-OHDA-exposed rat PD models by repressing pyroptosis, where proinflammatory signals associated with inflammation induce cell death [204].

In addition, meth induces the reduction of mitochondrial cytochrome c, anti-apoptotic Bcl2/BAX ratio along with a decrease in mitochondrial membrane potential, increases mitochondrial mass, enhances protein nitrosylation and diminishes protein levels of complexes I, III, and IV of the electron transport chain [205].

Lipocalin-2 (LCN2), which overexpression is involved in cell death in the adult brain, has been shown to be upregulated in hippocampal astrocytes as well as in the serum and CSF after meth exposure, with the PERK-mediated signalling pathway being involved in meth-induced LCN2-mediated mitochondrial-related neuronal apoptosis [206]. Similarly, LCN2 protein amounts were shown to be increased in the substantia nigra [207] and in the serum [208] of PD patients.

Uncoupling protein 2 (UCP2), a mitochondrial anion carrier ubiquitously expressed in many cell types to reduce oxidative stress, has been proposed as a therapeutic factor for modifying the progression of PD pathogenesis since its expression has been shown to attenuate rotenone-induced mitochondrial fragmentation [209]. Interestingly, although a direct effect of meth on UPC2 remains to be proven, the fact that meth causes a dysfunction in the respiratory chain of the mitochondria and given the involvement of UCP2 in mitochondrial it has been hypothesized that UCP2 might represent a new therapeutic targets also for combating meth-induced neurotoxicity [210]. Moreover, meth-, human immunodeficiency virus (HIV) gp120- and Tat-exposed human primary neurons manifest an increase in DRP1-dependent mitochondrial fragmentation, neuronal degeneration, microtubule-associated protein 1 light chain 3 beta-II (LC3B-II) lipidation and induced sequestosome 1 (SQSTM1, p62) translocation to damaged mitochondria [211]. Finally, the accompanying increased ROS and the inhibition of autophagy flux further suggest that meth either alone or in combination with HIV proteins, causes significant mitochondrial damage and neuronal injury [211].

A synthesis of the data set concerning our current knowledge of the similarities between the endoplasmic reticulum and mitochondrial stress in PD and meth abuse is presented in Table **3**.

#### 4.1.5 Alteration of the Immune System, Neuroinflammation and Autophagy Dysregulation

One additional and recently established possible route of action through which meth could pertain to awry physiology in PD is the immune system. The current focus on immunomodulatory approaches to prevent or delay the onset of idiopathic PD [212] suggests that both genetic and environmental factors might, in some or the other way, enhance the risk for idiopathic PD *via* the immune system [213]. Interestingly, the expression of DA across several immune cell types illustrates the complex mechanisms through which immune cells respond to DA and how the aberrant production of this neurotransmitter might impact immune regulation [214] as the pro-inflammatory immune-mediated mechanisms are crucial to the progression and pathogenicity of PD [215].

PD-specific T cells provide important mechanistic insights into PD pathogenicity [216]. It has been previously reported that T cells from individuals with PD responded to the presence of α-synuclein to a much greater degree when compared to the control group [217] as α-synuclein is known to be involved in the activation of innate and adaptive immunity where it significantly affects the phenotype and function of both CNS and peripheral nervous system (PNS) immune cells [218].

In this context, several deleterious effects of meth on the immune system have been reported [219]. Firstly, meth, *via* a myriad of deleterious effects on the CNS and PNS, impacts the host immune system [220], which might be linked to the pathogenesis of neuropsychiatric disorders [221]. Secondly, the drug influences the adaptive immune response, which might facilitate the acquisition of diverse diseases [222]. Thirdly, it has been indicated that meth exposure results in altered T cell cycle entry and progression, which might strongly contribute to detrimental effects on the immune system [223]. Regarding the interaction between the immune system and neuroinflammatory responses, meth, *via* its action on the immune cells and microglia, triggers the release of proinflammatory mediators, causing neuroinflammation. These neuroinflammatory responses are suggested to be mediated *via* the activation of the innate immune Toll-like receptor 4 (TLR4) [224]. Moreover, along with TLR4, DA D3 receptor signaling has been reported in mice to regulate meth-mediated activation of mast cells, which act as effector cells in various immune responses [225]. This set of data suggests that meth-mediated alteration of the immune system could possibly be a catalyzer for PD.

Chronic neuroinflammation is one of the hallmarks of PD pathophysiology [226]. Tumour necrosis factor-alpha (TNF-α) is involved in neuroinflammatory and excitotoxic processes in many neurodegenerative diseases by potentiating glutamate-mediated cytotoxicity through an increase of the expression of Ca^+2^ permeable-α-amino-3-hydroxy-5-methyl-4-isoxazole propionic acid (AMPA) and NMDA receptors [227]. Moreover, TNF-α triggers the activation of the Ikappa-B (IκB) kinase (IKK)/NF-κB and MAPK/activator protein 1 (AP1) pathways, which are essential for the expression of proinflammatory cytokines and for the induction of many biological events occurring downstream of TNF-α, including apoptosis and necrosis [228]. It has been established that TNF-α levels are significantly increased in the brains of PD patients [229], and post-mortem analyses of human PD or experimental animal models of the disease indicate activation of glial cells and an increase in pro-inflammatory factor levels, which supposedly play vital roles in the degeneration of dopaminergic neurons [230]. Indeed, the chronic release of proinflammatory cytokines by activated astrocytes and microglia leads to the exacerbation of dopaminergic neuron degeneration in the SNpc [231]. In this context, it is interesting to note that meth exposure activates neuroinflammatory cascades in the brain, and such neuroinflammatory processes in the striatum may underlie cognitive deficits, depression and Parkinsonism reported in meth addicts [232]. At the molecular level, it has been evidenced that meth triggers the expression and the release of TNF-α [233, 234]. Considering interleukins, increased levels of IL-6 have been evidenced in the nigrostriatal region and cerebrospinal fluid of PD patients [235], while in patients with meth-associated psychosis, IL-6 and IL-8 are significantly increased in correlation with the severity of the cognitive dysfunctions [236].

Dendritic migratory cells are major agitators of immune responses and inflammation that have been shown to trigger an autoimmune response, thereby establishing a potential link between Parkinsonism and autoimmunity [237]. Of interest were the observation that meth modulates the expression of a number of proteins by affecting immature dendritic cells [238] and that neurodegeneration seen following acute administration of meth was suggestive of being associated with the induction of cyclooxygenase-2 (COX-2), which causes a neuroinflammatory process that results in deleterious events in the cell [239]. Because COX-2 directly contributes to neuronal vulnerability, plays a key role in inflammation and is associated with the pathogenesis of PD [240], one can suppose a COX-2-mediated pro-PD action of meth.

As underlined in an earlier paragraph, the ionotropic purinoceptor P2X7 (P2X7R) has the ability to modulate proinflammatory signaling and promote neurodegeneration [241]. Furthermore, the involvement of neuronal P2X7 receptors in PD has been evidenced [242], and a study carried out in a Chinese population revealed that the P2X7 gene is also associated with the risk of developing late-onset sporadic PD [243]. In this context, the demonstration that meth stimulates microglial activation through P2X7R signaling [244] makes this class of receptors a possible vector for the drug to subserve the development of PD.

Moreover, PD patients display an imbalanced hypothalamic-pituitary-adrenal axis (HPA) and significantly increased cortisol levels, implying that the deregulation of glucocorticoid function may play a major role in the inflammatory processes observed in PD [245]. Indeed, meth, which has been effectively shown to induce significant alterations in the function of the HPA axis [246], could eventually favour PD *via* the deregulation of glucocorticoid-dependent inflammation. A recent investigation in human cerebral organoids using single-cell RNA sequencing (scRNA-seq) revealed that meth upregulates immune responses, complement factors and apoptosis with a marked alteration in cytokine gene expression, thus inducing profound neuroinflammatory changes [247]. Moreover, fMRI and blood analyses of meth-dependent human abusers demonstrated both structural and physiological alterations, as shown by an increased white matter volume in the superior and medial frontal gyri and left/right middle temporal gyrus and an augmentation of S100B and TNFα levels affirming an activation of neuroinflammation [248]. Finally, it has been hypothesized that prodromal PD involves gut inflammation and that the accumulation of toxic proteins partially causes degeneration of dopaminergic projections when transported from the enteric nervous system to the CNS. Supporting this notion, an interesting observation has revealed that the gut and brain profile in pre-motor and early-stage PD resembles the self-administration of meth in rats [249].

There exists a complex relationship between autophagy and inflammation, which includes both suppressive and inducible mechanisms, and some studies have suggested that modulation of one might lead to therapeutic interventions for diseases associated with the other [250]. Autophagy is a naturally regulated catabolic mechanism that allows the orderly degradation and recycling of cellular components, and that has been associated with PD pathogenesis [251]. The existence of a relationship among several PD-related genes, autophagy and mitochondrial dysfunction [252], the complex interplay of mutated genes in the autophagy-lysosomal pathway and the increased risk for developing PD along with an aberrant regulation of autophagy associated with the aggregation of α-synuclein observed in PD brain tissue [253], altogether mark the importance of autophagy regulation in PD. Moreover, it has been hypothesized that exosomes can transfer pathological α-synuclein from neurons to astrocytes. Thus, using animal and cell line coculture models, it has been demonstrated that exosomes isolated from meth-treated SH-SY5Y cells contained pathological α-synuclein and that the drug can significantly induce its aggregation and inflammatory responses in cultured astrocytes [254]. Chaperone-mediated autophagy is a process that can effectively and selectively degrade cytosolic proteins in lysosomes without vesicle formation. Because its activity declines in PD [255] and is decreased after meth exposure in neurons [256], this process is likely to play a pivotal role in meth-induced PD-like neurotoxicity.

The relationship between meth toxicity and mechanisms associated with autophagy [257], especially its influence on apoptotic autophagy of dopaminergic neurons [258], led to the hypothesis that meth, by deregulating autophagic mechanisms, edges the pathology of PD. Indeed, it has been evidenced that meth increases the levels of the autophagy-related protein markers, microtubule-associated proteins 1A/1B light chain 3B (LC3) and Beclin 1 in rat brain as well as in rat primary cultured neurons and in PC12 cells [259] and that the acute meth-dependent early increase in Beclin 1 and LC3 recruitment is mediated through the inactivation of the Akt/mammalian target of rapamycin (mTOR)/p70S6K pathway [260]. Additionally, overexpression of autophagy-related protein 5 (Atg5) and LC3 protein has been reported in the PFC of post-mortem cases of chronic meth users [261]. It is remarkable to observe that the factors cited above are also involved in PD. Thus, dysregulation of the mTOR pathway is a critical event in PD pathogenesis [262], while LC3 takes part in autophagosome buildup and Lewy bodies formation in the substantia nigra of PD brains [263]. In addition, variations in the genetic profile and expression of Atg5 have also been associated with the disease [264, 265].

However, things are not as simple as they seem since Beclin 1 is implicated in both autophagy and apoptosis and can either trigger or inhibit autophagy, depending on the proteins it interacts with [266]. For this reason, increasing its levels can theoretically produce both beneficial and deleterious effects. On the one hand, it has been shown that Beclin 1 plays an important role in the intracellular degradation of α-synuclein either directly or indirectly through the autophagy pathway and may present a novel therapeutic target for PD [267]. On the other hand, Beclin 1 can alter phagocytosis through an impairment of the recruitment of retromer to phagosomal membranes, the reduction of retromer levels and an impaired recycling of phagocytic receptors like a cluster of differentiation 36 (CD36) and triggering receptor expressed on myeloid cells 2 (TREM2) [268].

PKCδ is another key element in meth-induced autophagy, ubiquitin-proteasome system (UPS) dysfunction and cell death of mesencephalic dopaminergic neurons. Indeed, meth significantly increases PKCδ and caspase-3 activation, the accumulation of ubiquitin-positive aggregates and microtubule-associated LC3 levels [269]. As a matter of fact, because the meth-dependent increase in PKCδ expression in the striatum is accompanied by oxidative stress and dopaminergic damage, inhibition of PKCδ could serve to bring protection against meth-induced neurotoxicity [270]. This theory is further supported by the observation that PKCδ up-regulation impels the neuroinflammatory responses and dopaminergic neurodegeneration in experimental models of PD [271] and by the evidence that the inhibition of PKCδ transactivation offers neuroprotection in both cell cultures and animal models of PD [272].

Peli1 is an E3 ubiquitin ligase that acts as a positive regulator of inflammatory responses in microglia *via* the activation of NF-κB and MAPK and is substantially induced in the substantia nigra of the human and mouse PD brains [273]. As manifested in mouse brain and microglial cell cultures, meth significantly enhanced toll-like receptor (TLR) 4 and TIR-domain-containing adapter-inducing interferon-β (TRIF) expression, NF-kB and MAPK pathways activation and the production of interleukin (IL)-1β, TNF-α and IL-6, suggesting a concordant involvement of Peli1-mediated neuroinflammation induced by meth [274]. Human PET investigations have been performed in order to explore microglial changes in chronic meth users, and scans, using the radiotracer (C-11) (R)-PK11195 have reported a massive increase in the 18 kDa translocator protein (TSPO), a biomarker for microgliosis, in such abusers [275], although the use of the second-generation TSPO radioligand (F-18) FEPPA did not confirm this data [276]. Herein, a recent study has revealed TREM1-and TSPO-PET tracers as propitious means to investigate cell types involved in immune reactions in order to characterize the clinical potential pertaining to PD rodent models and human postmortem tissue [277].

Overall, it therefore appears that there is a consequent homology with regard to the mechanisms implied in the alteration of the immune system, neuroinflammation, and autophagy dysregulation occurring in Parkinson's disease and triggered by methamphetamine abuse (Table **4**).

#### 4.1.6 Apoptosis

Apoptosis has been largely evidenced as the main mechanism of neuronal death in PD [278], and several enthralling theories have shown that multiple molecular pathways are involved in the propagation of PD pathogenesis, with autophagy and apoptosis being the two key cellular death pathways that can be targeted as possible therapies aimed at combating this disease [279]. As a matter of fact, this complex disorder engages different biological interactions that collectively lead to neural cell death. These interactions encompass, among others, the dopaminergic pathway, the mitochondrial pathway and the p53-DNA damage pathway. Indeed, p53 is at the centre of multiple signalling cascades involved in the aetiology of PD [280] and interestingly plays a crucial role in the long-term deleterious effects of meth on dopaminergic terminals and cell bodies as its knock-out insinuates protection against drugs like meth that act on brain DA systems [281].

As part of the complex p53-containing network, the PD-associated parkin lowers p53 mRNA levels and represses p53 promoter transactivation, and its depletion enhances p53 expression and mRNA levels in fibroblasts and mouse brains [282]. Moreover, the p53 upregulated modulator of apoptosis (PUMA) expression is significantly involved in the apoptotic mechanisms taking place in PD [283, 284]. It is a dominant regulator of oxidative stress by inducing Bax activation and neuronal apoptosis [285]. In addition, mutations in PINK1 have been linked to the occurrence of early onset Parkinsonism [286], and an increase in PINK1 protein might be an intrinsic protective mechanism to limit cellular death. Finally, the autophagosome regulator Beclin 1 plays an important role in the intracellular degradation of α-synuclein either directly or indirectly through the autophagy pathway and may present a novel therapeutic target for Lewy body disease and PD [267].

In this general context, beyond the obvious capability of meth to induce apoptosis [287], the drug has been shown to interfere with several PD-associated pathways. Firstly, it strongly affects autophagy and mitochondrial integrity and induces apoptosis, with functional but not non-functional PINK1 being able to reverse these phenomena [288]. Secondly, the pro-apoptotic caspase11, which mediates dopaminergic cell death in a PD mouse model [289], also plays essential role in meth-induced dopaminergic neuronal apoptosis [290]. Thirdly, meth increases the expressions of NUPR1, CHOP, p53 and PUMA [291] to mediate endothelial cell apoptosis through the NUPR1-CHOP/p53-PUMA/ Beclin 1 signalling pathway, where Beclin 1 is involved in meth-mediated autophagy [292], an observation recently evidenced in post-mortem human brains of chronic meth abusers [171].

Conferring various other mechanisms *via* which meth induces apoptosis, it is noteworthy that such mechanisms are discernibly involved in PD pathogenesis. It has been shown that the expression of miR-133b is depressed in cellular models of PD, as shown by the demonstration that overexpression of miR-133b inhibits cellular apoptosis by regulating the extracellular signal-regulated protein kinase (ERK1/2) signalling pathway in MPP+ induced PC12 cells [293]. The additional observation that meth induces a significant decrement in miR-133b expression at the transcriptional level in PC12 cell cultures is a clear-cut indication of how meth could possibly induce apoptosis by altering miR-133b [294].

The CCAAT-enhancer binding protein (C/EBPβ) and the insulin-like growth factor binding protein (IGFBP5) are important regulators of cellular apoptosis. Intra-striatal high-dose meth administration in rat brain and neuronal cells triggers an IGFBP5-mediated, PUMA-related mitochondrial apoptotic signalling pathway and increases C/EBPβ protein expression accompanied by an augmentation of neuronal apoptosis and autophagy [295]. In this context, an interesting recent investigation revealed that C/EBPβ/δ, by regulating transcription and proteolytic cleavage of α-synuclein and monoamine oxidase (MAO) B, mediates PD pathogenesis [296].

Moreover, the compulsive intake of meth in rats is accompanied by an immense increase in the autophagy biomarkers Unc-51. Like Autophagy Activating Kinase 1 (ULK1) and phospho-Beclin1, a significant increase in the mRNA levels of autophagy-related genes including Atg2a, Atg5, Atg14, and Atg16L1 together with an associated augmentation in the expressions of p53, caspases 6 and 9 and an obvious decrease in anti-apoptotic B-cell lymphoma 2 (Bcl2) protein [297]. Recent *in vitro* and *in vivo* investigations have also shown that the phosphorylation of α-synuclein is significantly increased after meth treatment with phosphorylation of α-synuclein at S129, a well-established hallmark of PD [298], exacerbating its aggregation and triggering meth-induced neurotoxicity and apoptosis [299].

Finally, the isoform protein kinase C delta (PKCδ) has been evidenced as a key mediator in inducing apoptotic cell death in PD models, and its suppression effectively blocks apoptotic processes in such models [272]. Moreover, it has been established that the caspase-3-dependent proteolytic activation of PKC increases MPP(+)-induced apoptosis in dopaminergic neuronal cells [300] and that, conversely, its suppression prevents this phenomenon in the same model [301]. In this context, PKCδ has been evidenced as a critical target gene involved in dopaminergic neurotoxicity and degeneration induced by meth [302], and an animal study carried out in wild-type C57BL/6 and p47 phosphate-repressible alkaline phosphatase (*phox)* knockout mice models revealed that meth significantly increased the expression of PKCδ, which is also an important regulator for p47phox activation induced by meth [303].

A summary of the apoptotic mechanisms/factors implicated in both PD and meth abuse is presented in Table **5**.

#### 4.1.7 Deregulation of Neurotransmitter Homeostasis

The interactions of neurotransmitters with their receptors intercept how strongly neurons respond to further relay any information within the brain circuits, and this complex interactive network is maintained through intricate homeostatic mechanisms. The neuropathology of PD is characterized by a disruption of the neurotransmitters system, such as dopaminergic, serotonergic, noradrenergic, glutamatergic, cholinergic, and GABAergic transmission, along with an alteration of their receptors and transporters [304, 305]. Importantly, meth exposures have been associated with changes in neurotransmitter levels in several central brain regions, with prolonged use of meth causing a cascade of neurochemical imbalances that result in long-term brain and neuronal damage.

##### 4.1.7.1 Dopaminergic Transmission Alteration

The breakdown of the dopaminergic system is a well-characterized major feature of PD that has been previously extensively described and reviewed by others [306], and that will not be the subject of an exhaustive description here. Nonetheless, it should be noted that *in-utero* meth exposure triggers changes in the mesolimbic dopaminergic system that becomes more sensitive to the administration of an acute dose of the drug in adulthood, thereby indicating that offspring exposed to meth before birth could be more sensitive to meth at adulthood [307]. Moreover, the fact that meth induces long-lasting damage to nigrostriatal DA neurons *via* oxidative stress supports the hypothesis that PD development could be induced by meth as a result of oxidative damage during development leading to an age-related change in the neurotrophic capacity of the striatal DA system [308]. This hypothesis has gained more weight with the subsequent demonstration of a dose-dependent effect of meth on DA and BDNF levels [309].

At the molecular level, it has been shown that meth perturbs dopaminergic transmission through its binding to DAT, which blocks the reuptake of DA [310]. More recently, it has been evidenced that methamphetamine induces dopaminergic damage *via* a D1 receptor-mediated activation of autophagy [311]. In addition, meth modulation of DA neurotransmission and resulting behavioral responses is, in part, due to the meth-dependent regulation of Ca^2+^-activated potassium channel activity [312]. As dopaminergic neurons display large cytosolic Ca^2+^ oscillations linked to fundamental mitochondrial oxidative stress and susceptibleness in aging and PD [313], meth-induced vulnerability for calcium-induced neurodegenerative processes is likely to occur. As a whole, the meth-induced perturbation of the dopaminergic transmission leads to a loss of hippocampal function, which results in memory and learning deficits [31].

Apart from the dopaminergic system, other neurotransmitters are most likely to be involved in PD pathogenesis since treatments solely focused on DA regulation are unable to alleviate both motor and non-motor symptoms, particularly those that develop at the early stages of the disease [314]. Moreover, the imbalance of other neurotransmitters accounts for the heterogeneity and complexity of the neuropsychiatric symptoms observed in PD patients [315]. The following sections discuss the neurotransmitter systems affected in PD and altered by meth.

##### 4.1.7.2 Cholinergic Deficits

Cholinergic system degeneration not only contributes to cognitive deterioration but also to other non-motor features and motor impairments in PD associated with a widespread loss of cholinergic nicotinic and muscarinic receptors [316-318]. This cholinergic denervation occurs in PD and is often more severe than in AD [319], with the cortical cholinergic network beginning to degenerate early in the disease process of PD [320]. The presence of a Lewy body in neurons of the nucleus basalis of Meynert, which is the source of cholinergic innervation of the cerebral cortex, basal forebrain cholinergic system degeneration and cholinergic denervation, probably due to degeneration of brainstem pedunculopontine nucleus neurons, is suggestive of an involvement of a collapse of the cholinergic system in PD [321]. Moreover, cholinergic dysfunction contributes to mobility deficits in PD, and the specific loss of nigral excitation of cholinergic interneurons contributes to Parkinsonian motor impairments [322].

Muscarinic acetylcholine receptors (mAChRs) can be found in the neocortex, hippocampus, olfactory tubercle and amygdala, but the highest concentration is found in the striatum. In this context, meth-exposed mice show impaired novel object recognition and an increased number of mAChRs in the hippocampus. Thus, the cholinergic system might play an important role in long-term meth-induced cognitive deficits in adulthood [323]. In the same manner, nicotinic AChRs (nAChRs) are involved in meth and 3,4-methylenedioxymethamphetamine (MDMA)-induced neurotoxicity [324] and also in dopaminergic damage caused by repeated and high doses of meth [325], which also alters α4β2 and α6β2 nAChRs expression in rats [326].

The degeneration of brain cholinergic systems contributes to gait-balance alterations and attentional deficits in PD patients where stimulation of α4β2 nAChR enhances attention and improves gait-balance function [327], and α4β2 nAChR is also an important therapeutic target in meth-induced alterations in cognitive functions like decision-making [328]. It has been shown that amphetamine enantiomers bind to the homomeric α7 nicotinic acetylcholine receptor (α7 nAChR) and competitively inhibit acetylcholine responses, which clearly suggests that stimulants similar to amphetamines might involve these receptors in mediating their cholinergic effects observed in substance abuse disorders [329].

The bidirectional interaction between acetylcholine and DA signaling in the striatum is critical, and alteration in their ratios are eminent in PD since cholinergic interneurons express DA receptors and dopaminergic neurons express both muscarinic and nicotinic receptors. Therefore, drugs that increase striatal DA release can potentiate both DA and acetylcholine release [330]. The brainstem reticular formation represents the archaic core of pathways that connect the spinal cord and the encephalon. It is involved in autonomic, motor, sensory, behavioral, cognitive, and mood-related functions. The recognition that drugs like amphetamine and meth affect the release of DA and acetylcholine from this brain area further denotes how meth affects the functions mentioned above [331]. Concerning the DA and acetylcholine muscarinic receptors, autoradiographic assessment revealed that prenatal and adult meth exposures in rats decrease the expression of muscarinic receptors (M1, M2) in the caudate-putamen, dorsal hippocampus, CA1, CA3 and dentate gyrus and reduced D1 DA receptors in the motor cortex and substantia nigra [332].

Changes in the cholinergic system are thought to contribute to PD complications, including cognitive difficulties, postural instability and sleep disturbances, and the heterogeneity of cholinergic degeneration in PD posits a challenge to assess the acetylcholinergic receptors as therapeutic targets. Since some studies suggested that nigrostriatal damage affects nicotinic receptor-mediated dopaminergic signalling, therapeutic modulation of the nicotinic cholinergic system might offer novel therapeutic approaches to manage PD [333]. In this context, it has been reported some overlapping effects by meth and nicotine as nicotinic agonists substituted for meth-like effects [334].

##### 4.1.7.3 Glutamatergic/GABAergic Imbalance

Although DA has been at the focal point of neurotransmitters involved in PD, glutamate and gamma-aminobutyric acid (GABA), respectively excitatory and inhibitory neurotransmitters, are also affected by DA homeostasis in controlling neural activity in PD pathogenesis [335] with alterations in the GABAergic and glutamatergic neurotransmission contributing to the axial symptoms of the disease [336]. Indeed, lower concentrations of GABA and glutamate have been observed in PD patients when compared with normal subjects [337]. Moreover, abnormal synaptic signaling caused by an enhancement of extracellular glutamate results in neuronal excitotoxicity and death linked to an impaired ability of glial cells to reuptake and respond to glutamate, a phenomenon common in PD [338]. Such increased concentrations of extracellular glutamate inhibit cystine uptake, which leads to glutathione depletion and PD-associated oxidative glutamate toxicity [339]. Interestingly, GABAergic and glutamatergic neurotransmitter systems are critical in the pathophysiology of addiction, as illustrated by the fact that meth exposure limited to the prenatal phase has strong effects on the GABAergic and glutamatergic systems in the adult rat brain [340]. Additionally, meth elicits an augmentation of endogenous glutamate in the brain, partially explaining meth-induced memory deficits [341]. Moreover, prior exposure to meth induces a faster escalation of meth self-administration with consequent alterations in hippocampal glutamate AMPA receptor mRNAs in rats [342]. Proton magnetic resonance spectroscopy analysis has recently demonstrated that cognitive deficits in individuals with meth dependence might be related to alterations in the levels of GABA and glutamate/glutamine in the PFC [343]. In line with this, studies carried out in rats have shown that meth alters the levels of DA, serotonin, glutamine and glutamate [344] as well as the levels of noradrenaline and GABA in the PFC and most predominantly in the hippocampus [345].

It is also important to note here that meth administration causes expression changes in neurotoxicity-associated signaling cascades and significant atrophy of the PFC [346], thus triggering cognitive dysfunctions similar to those observed in PD patients [347]. Psychotic symptoms are common in PD [348], and many PD patients experience neuropsychiatric disturbances such as depression, psychosis and behavioral and cognitive changes [349]. Given the nature of the cognitive sensations that are associated with the mesolimbic pathway, it is involved in conditions such as addiction and depression. In this context, it has been shown that meth increases glutamatergic signals to the cortex from both the nigrostriatal, as well as the mesolimbic reward circuits, and augments DAergic signals from the mesocortical pathway, which affect the GABAergic interneurons, ultimately leading to a dysregulation of the signals and causing psychotic symptoms during meth intoxication [350].

Additionally, deficiency in glutamate transporter-1 (GLT-1), which is mainly responsible for the clearance of glutamate at the synapse, including DA synapses, was associated with Parkinsonian phenotypes. Indeed, progressive motor deficits and nigral DA neuronal death have been observed in mice, accompanied by the presence of reactive astrocytes and microglia in the SNpc [351]. Related to this fact, excitatory signaling and glutamate homeostasis are well-known pathophysiological substrates underlying addiction-related behaviors spanning multiple types of psychostimulants where considerable interest has focused on GLT-1 [352].

Furthermore, the mGluR5-calcium-dependent cascade causes axonal degeneration, and henceforth the mGluR5 antagonists provide effective therapy to prevent the disease process of PD [353]. Concerning meth, it is interesting to note that mGluR5 receptors mediate meth reinforcement and meth-seeking behaviour [354] and that pharmacological inhibitors of mGluR5 receptor function may represent a novel class of potential therapeutic agents for the treatment of meth addiction [355].

Several additional evidences of the involvement of meth in the disruption of the GABAergic system have also been reported. Firstly, manipulation of glutamatergic and GABA-ergic systems in the shell-nucleus accumbens modulates meth-induced enhancement of LTP in the hippocampus, which perhaps occurs due to cross-talk between nucleus accumbens and hippocampus as stimulation of the nucleus accumbens has been shown to alter hippocampal plasticity [356]. Secondly, it has been shown that meth exposure promotes microgliosis and inflammation *via* astrocytic glutamate release in co-cultures of primary neurons and microglia [357]. Thirdly, escalating doses of meth alters the NMDA and AMPA glutamate receptor subunits in the striatum and frontal cortex of rats, which partly also explains the mnemonic deficits and psychotic behavior associated with meth abuse [358] with such meth-induced neuroadaptations at glutamatergic synapses being under the control of multiple epigenetic regulation [359].

Since the GABAergic system is involved in amphetamine-type stimulant use disorders [360] and because GABBR1 is associated with meth use disorder and relapse [361], GABBR1 might represent a pivotal factor linking meth abuse to PD pathogenesis.

The studies have evidenced some mechanisms responsible for neurotransmission defects which are common to PD pathogenesis and meth abuse, as presented in Table **6**.

#### 4.1.8 Glycogen Synthase Kinase-3 Beta (GSK3β) Hyper Activation

GSK3β is a serine/threonine protein kinase involved in multiple neuronal functions such as neurogenesis, neurotransmission and synaptogenesis but also plays key roles in multiple cellular processes accounting for the progression of numerous diseases. Thus, dysregulation of GSK3β has been implicated in nigral dopaminergic neurodegeneration [362], and its inhibition, following a consistent number of observations, has been increasingly considered as an anti-PD therapy [363].

Firstly, the gene encoding GSK3β has been linked to PD risk [364-366] and genome-wide studies have established α-synuclein and tau genes as two of the most important factors in the genesis of PD with GSK3β contributing to both α-synuclein and tau phosphorylation [367]. Secondly, LRRK2, which is involved in familial forms of PD, can directly interact with and activate GSK3β, resulting in increased phospho-tau formation [368, 369]. Likewise, *6-hydroxydopamine* (6-OHDA)-induced *in vitro* and *in vivo* models of PD have demonstrated elevated levels of LRRK2 and GSK3β, the two kinases directly involved in the formation of tau and α-synuclein proteins, causing PD [370]. Thirdly, elevated tauopathy in the striatum of both PD and Parkinson’s disease dementia (PDD) has been found to correlate with increased levels of phosphorylated GSK3β [371]. Fourthly, MPP^+^/ MPTP treatment activates GSK3β and mediates tau phosphorylation, which is dependent on α-synuclein in Parkinsonism models such as SH-SY5Y co-transfected cells, mesencephalic neurons, transgenic mice overexpressing α-synuclein, and post-mortem striatum of PD patients [372]. Finally, tau has been identified as a susceptibility factor for Parkinson's [45] and PET scan analysis of Parkinsonian tauopathies such as progressive supranuclear palsy, corticobasal degeneration, frontotemporal dementia and parkinsonism linked to chromosome 17 have revealed that there is a propensity of brain areas to bind to pathological tau [373].

GSK3β, along with Cdk5, is involved in the abnormal hyperphosphorylation of tau and analysis of their single nucleotide polymorphisms revealed that they play an important role in determining the risk profile for PD [374]. Moreover, it has been shown that GSK3β facilitates apoptotic conditions and that its inhibition protects the dopaminergic neurons from various stress-induced injuries, thereby indicating a probable involvement of GSK3β in PD pathogenesis [375]. Interestingly, a recent investigation carried out on serum of PD patients, in brain tissues of MPTP-induced mice and in 1-methyl-4-phenylpyridinium (MPP^+^)-induced SH-SY5Y neuroblastoma cells has reported that cellular apoptosis could possibly be repressed by targeting the Akt-mediated GSK3β/β-catenin signalling pathway [376]. Moreover, overexpression of GSK3β was shown to decrease antioxidant defense processes due to its involvement in Nrf2 regulation and, for this very reason, are crucial targets for PD therapeutics [377]. Finally, GSK3β is a substrate of the Skp1-Cul1-F box protein-7 (SCF^Fbxo7/PARK15^) ubiquitin ligase [378], which deficiency was associated with early-onset PD [379].

Back to meth and considering the above-described robust connexion between GSK3 biology and PD pathogenesis, it is important to note that GSK3β interaction with α-synuclein is itself a very crucial nexus mediating meth-induced neurotoxicity, which leads to the blockage of autophagy-lysosomal degradation pathway and eventually to cellular apoptosis [258]. Moreover, meth exposure increases GSK3β activity and decreases excitatory synapse density in the hippocampus of adult mice [380], with similar effects having been noticed in human neuroblastoma cells where meth treatment increases tau phosphorylation and GSK3β activity [381].

Moreover, meth increases GSK3β activity by downregulating its phosphorylated levels in the rat hippocampus [382], while decreased p-GSK3β levels further disturb insulin signaling [381], which may play a role in PD pathogenesis. It has also been demonstrated that GSK3β activity is increased in the nucleus accumbens [383] and in the ventral tegmental area [384], an area involved in PD pathogenesis. Indeed, chronic meth administration in rats decreases the levels of phosphorylated GSK3β at Ser 9 in the nucleus accumbens [385]. Finally, acute meth treatment decreases the levels of phosphorylated GSK3β [386], a finding consistent with the fact that amphetamine induces a decrease in the phosphorylation of GSK3β in the mouse striatum [387].

#### 4.1.9 Neurogenesis

##### 4.1.9.1 Neurogenesis Alteration in PD

Alterations in adult neurogenesis appear to be a common hallmark of several neurodegenerative diseases, including PD, AD as well as Huntington's disease (HD) [388]. More specifically, considering PD, adult neurogenesis is severely affected [389], and impairments in stem cell proliferation, differentiation and survival, as well as neurite outgrowth, significantly contribute to the pathogenesis of the disease [390]. Moreover, the density of nestin and tubulin-positive cells was found to be reduced in the dentate gyrus of PD patients [391], and cognitive deficits in PD have been implicated in cholinergic and noradrenergic dysfunction involving hippocampal functions [392].

PD is accompanied by a deficiency of neural stem cells (NSCs) pool in the affected brain regions. Therefore, cell replacement therapy has emerged as a promising restorative therapy for PD patients [393]. A detailed examination of neurogenesis in the post-mortem brains of PD patients has reported a reduction in the number of proliferating cells in the subventricular zone (SVZ) as a consequence of dopaminergic denervation [391].

Hippocampal dysfunction is common in PD patients and likely contributes to cognitive impairment [394], which correlates with the degree of dementia [395]. Also, in the human hippocampus, the levels of endogenous α-synuclein are increased in Lewy Body Dementia (LBD) and the numbers of (SRY-sex determining region Y)-Box Transcription Factor 2) (SOX2)-positive cells are decreased [396], a phenomenon that triggers neurodegeneration and impaired neurogenesis in the adult mouse brain [397].

Dopamine depletion, as well as the accumulation of α-synuclein, as PD-related pathogenic factors, also have an impact on adult hippocampal neurogenesis. Thus, there exists a pernicious synergistic interplay between α-synuclein modification and DA depletion, which further contributes to impaired neurogenesis in PD [398], and the degree and temporospatial dynamics of adult olfactory bulb neurogenesis are modulated by α-synuclein in transgenic mice [399]. At the molecular level, the accumulation of α-synuclein impairs neurogenesis by reducing neural progenitor cells (NPCs) survival *via* the down-regulation of Notch-1 expression [400]. Notch1 signalling maintains stem cell self-renewal, proliferation, neuronal differentiation, and glial determination [401, 402], plays an important role in adult neurogenesis in the hippocampus as it regulates proliferation in the adult dentate gyrus [403] and supports the survival of both progenitors and newly differentiating cells in the developing nervous system [404]. Interestingly, it has been shown that depletion of DA in rodents decreases precursor cell proliferation in the SVZ and that the number of proliferating cells in the SVZ is reduced in the post-mortem brains of individuals with PD [391, 405]. Moreover, α-synuclein directly binds to the vicinity of the Notch1 promoter and also interacts with the p53 protein to facilitate or increase Notch1 signalling repression and impair the maturation and survival of NPCs, thereby providing a molecular basis for α-synuclein-mediated disruption of adult neurogenesis in PD [406].

##### 4.1.9.2 Specific Meth/PD Links Regarding Neurogenesis

It has been evidenced that meth can alter adult hippocampal neurogenesis by decreasing NPCs proliferation and survival *via* excessive protein nitration [407]. In addition, an interesting link between adult hippocampal neurogenesis and meth addiction has uncovered a mechanistic relationship between neurogenesis and drug seeking, where abstinence from meth addiction enhances the proliferation and differentiation of neural progenitors and increases adult neurogenesis in the dentate gyrus [408]. Moreover, several factors involved in neurogenesis were shown to be similarly affected in PD and under meth abuse conditions.

Nuclear receptor related 1 (NURR1), a nuclear receptor guiding midbrain dopaminergic neuron development, is critical for the survival and maintenance of dopaminergic neurons and has been implicated in dopaminergic neuron-related disorders. Identifying NURR1 mutation in PD patients suggested that NURR1 plays a regulatory role in the development of DA neurons [409]. In addition, overexpression of NURR1 was found to enhance the ability of mouse NSCs to differentiate into DA neurons in PD rat models [410] and a similar effect was observed with NURR1 agonist [411]. Recently, it has been shown in α-synuclein transgenic mice that loss-of-function mutations in NURR1 are associated with familial PD [412]. In this context, acute meth administration significantly increases the levels of NURR1 mRNA in the pre-limbic, primary motor and primary somatosensory cortices and VTA [413], while chronic meth exposure decreases NURR1 expression [254] with reduced NURR1 levels exacerbating meth-induced acute and long-term toxicity in adult mice [414]. Moreover, considering that C-myc, an important adult neurogenesis-regulating factor, is increased in reactive astrocytes of the substantia nigra of PD patients [415], it is worth noting that meth upregulates C-myc at both the mRNA and protein levels in the mouse brain [416]. In the same manner, given that mutations in the parkin gene are common in early-onset and familial PD and that parkin expression has an inverse relation with N-myc levels in the developing mouse and human brains and in human neuroblastoma cell lines [417] and considering that N-myc is increased in reactive astrocytes of the substantia nigra of PD patients [415], N-myc is interestingly also associated with the neurotoxic process induced by meth that also increases COX1, which is linked to meth-induced DA neuronal injury expression in the ventral midbrain [418].

In addition to its importance during development, the transcription factor pituitary homeobox 3 (Pitx3) also has roles in the long-term survival and maintenance of the midbrain DA neurons [419]. Interestingly, chronic meth administration causes differential regulation of Pitx3 in the rat midbrain [420]. Moreover, overexpression of the peroxisome proliferator-activated receptor-gamma coactivator PGC-1alpha *(PGC-1α)* results in DA depletion associated with lower levels of Pitx3. It enhances susceptibility to MPTP [421], which suggests that neuroprotective strategies should be targeted at PGC-1α in PD. NURR1 and Pitx3 are required for the expression of several genes encoding proteins that determine mature midbrain DA neuron identities, such as tyrosine hydroxylase (TH), dopamine transporter (DAT), vesicular monoamine transporter 2 (VMAT2), aromatic l-amino acid decarboxylase (AADC) and DA receptor D2 (DRD2) [422]. In this context, meth decreases the expression of TH [423], DAT [424] and VMAT2 levels [425]. Importantly, VMAT2 is significantly reduced in the brain of PD patients [426], and its upregulation protects against meth toxicity [427].

Wnt/β-catenin signaling plays a vital role in adult neurogenesis. It is required for the specification and neurogenesis of midbrain dopaminergic neurons [428], which degenerate in PD and MPTP mouse model of PD [429], while pertinent levels of Wnt signaling are also essential to improve the cell replacement therapy for PD [430]. Meth downregulates Wnt/β-catenin signaling [431] and induces the expression of Dickkopf WNT Signalling Pathway Inhibitor 1 (DKK1) [432], a neurodegenerative factor that serves as an antagonist of the canonical Wnt signaling pathway, but that simultaneously induces pro-survival Wnt/β-catenin signaling in hippocampal neurons. Indeed, while expression of DKK1 is required for proper neural development, overexpression of DKK1 is one characteristic of neurodegenerative diseases, including PD [433].

Anxiety disturbances are recognized as common psychiatric comorbidities in PD and contribute to significant impairments in areas of cognitive, functional, motor, and social performance [434]. A study in transgenic animals demonstrated that an impairment in adult hippocampal neurogenesis strikingly increased anxiety-related behaviors [435], while a functional association between adult neurogenesis and stress-induced anxiety- and depressive-like behaviors has been evidenced [436]. Indeed, neurogenesis differentially affects behavior as increasing adult hippocampal neurogenesis can affect anxiety and depression-related behavior through a mechanism independent of the hypothalamic-pituitary-adrenal (HPA) axis. Interestingly, while Bcl2-associated X (Bax) ablation prevents dopaminergic neurodegeneration in a mouse model of PD [437], the use of future techniques to specifically inhibit Bax in the hippocampus could be used to augment adult neurogenesis and to decrease PD-associated anxiety-like behaviors [438]. In this context, research conducted on both humans and a genetic mouse model characterized by high meth ingestion has demonstrated that its abuse leads to immune dysfunction and neuropsychiatric impairment accompanied by anxiety-like behavior [439]. Moreover, in relation to meth and Bcl2-associated X (Bax) apoptosis regulator, it has been demonstrated that meth induces an increase in Bax/Bcl-2 ratio in neuroblastoma cells [440], which implies that meth might cause impairment in neurogenesis *via* activating Bax.

Nrf2 is a transcriptional master regulator that not only maintains the redox homeostasis in cells by provoking the expression of antioxidant, anti-inflammatory and cytoprotective genes but also strongly influences NSCs function and fate determination by reducing the levels of ROS for the benefit of NSC survival and neurogenesis. Because Nrf2 is under the positive control of miR-7 [441], which is highly expressed in TH-positive dopaminergic neurons, it has been suggested as a putative therapeutic target in neurogenerative diseases like PD [442]. Interestingly, meth significantly downregulates Nrf2 expression in rats, thus exacerbating chronic nervous system toxicity [443]. In addition, a study conducted on hippocampal progenitor cells from adult rats has demonstrated that meth exposure decreases cell proliferation by upregulating the cell cycle regulators p53/p21 and promoting the accumulation of p21 in the nucleus [444]. In this context, it has been shown that cellular senescence is promoted *via* the upregulation of the p53/p21 pathway due to the G2019S most prevalent LRRK2 mutation, which, through an increase of its activity further, accelerates α-synuclein aggregation and contributes to PD progression [445].

Finally, all these elements, together with the fact that meth negatively impacts SOX2 [446], disturb DA homeostasis by decreasing its levels and activating α-synuclein to ascertain that meth-induced concurs to an alteration in adult neurogenesis as observed in PD.

Table **7** summarizes the parallels between PD pathogenesis and meth abuse regarding neurogenesis alteration.

### 4.2 The Gut and the Gut-brain Axis are Impacted in Parkinson's Disease and by Methamphetamine Abuse

The gut-brain axis allows two-way communication between the central nervous system and the enteric nervous system (ENS), thus linking the cognitive and emotional centers of the brain to the peripheral intestinal functions. The gut-brain axis is physically connected through millions of nerves, including the vagus nerve, is chemically associated with neurotransmitters such as serotonin and GABA, and is well connected to the immune system [447]. A substantial number of studies have prefigured that gut microbes might contour neural development and modulate neurotransmission, thereby contributing to the pathogenesis and/or progression of many neurodevelopmental, neuropsychiatric and neurological disorders, including PD [448, 449]. Indeed, alterations in gut-brain-microbiome interactions have been identified in several rodent models exhibiting digestive, psychiatric, and neurological disorders [450].

The gut microbiota and its metabolites have been suggested to be involved in the pathogenesis of PD by regulating neuroinflammation, barrier function, intestinal permeability and neurotransmitter activity. Thus, the microbiota-gut-brain axis provides a pathway for the interrelationships of the vagus nerve and the transmission of α-synuclein in the ENS [451, 452]. Moreover, α-synuclein misfolding commences at a very early stage of PD in the gut, supposedly induced by gut microbial toxins [453], and it has been observed that gut-to-brain propagation of pathologic α-synuclein occurs in a prion-like manner *via* the vagus nerve to cause PD [454, 455].

Accumulating evidence indicates that the emergence of gastrointestinal manifestations precedes both the onset of motor symptoms and the diagnosis of the disease, thus supporting the potential involvement of the microbiome-gut-brain axis in the underlying pathological mechanisms of PD [456], a hypothesis reinforced by the fact that the gut bacteria regulate movement disorders in a PD mouse model [457]. Another investigation carried out in MPTP-induced PD mice showed that motor impairment and the drop in striatal neurotransmitter levels were accompanied by gut microbiota perturbation and by an increase in the pro-inflammatory TLR4/ TBK1/NF-κB/TNF-α signaling pathway [458]. Finally, the intrinsic activity present in the gut microbiota is immensely involved in maintaining dopamine homeostasis by facilitating dopamine synthesis as well as its metabolite breakdown [459]. Therefore, any gut dysbiosis is expected to affect the dopamine bioavailability to enhance the vulnerability to develop PD.

Interestingly, the gut-brain axis plays a role in substance use disorders by affecting the brain's response to drugs [460, 461]. Indeed, psychoactive drugs like meth, which predominantly exert their primary effects within the CNS, are known to have abstruse effects on the gut microbiome, where the gut microbiota and its metabolites significantly affect reward and memory [462]. In a similar vein, meth intoxication has been shown to cause alterations in the diversity and taxonomic structure of the gut microbiome in mice [463] and to alter gut microbiota and induce depressive-like behavioral symptoms in rats [464]. In addition, escalating dose-multiple binge methamphetamine treatment in mice alters gut microbiota composition, elevates pathogenic bacteria with a simultaneous decrement of probiotics, and enhances intestinal inflammation [465]. Of importance is also the fact that meth can perturbate the gut-brain axis *via* an excessive production of pro-inflammatory cytokines, thus leading to a loss of the intestinal barrier integrity [466].

Another significant point is that meth exposure increases the levels of α-synuclein and decreases the levels of parkin and tyrosine hydroxylase in the myenteric plexus of rats [467]. This clearly indicates that gut biomarkers might provide eminent links between meth-induced toxicity in the gut and its correspondence to the increased susceptibility of developing PD later in life.

While PD-related gut microbiota dysbiosis has been associated with the impairment of the short-chain fatty acids (SCFAs) producing process, lipid metabolism, immunoregulatory function, and intestinal permeability [468], it has been shown that meth exposure decreases the expression of tight junction proteins zonula occludens-1 (ZO-1) and epithelial cell adhesion molecule (EpCAm) in the intestinal tissue of mice, where the presence of fatty acid-binding protein *1 (*FABP-1) in sera further suggests disruption of the gut wall [469].

Toll-like receptors play a crucial role in innate immunity, and dysregulation in their signaling may be implicated in α-synucleinopathy, such as in PD [470]. In this context, meth is known to activate microglia *via* the TLR4/Myelin differentiation factor 2(MD2) complex, thus modulating the abundant production of pro-inflammatory cytokines in the CNS [471]. Moreover, the nucleotide-binding oligomerization domain leucine-rich repeat and pyrin domain-containing protein 3 (NLRP3) inflammasome acts as a key player in both coordinating the host physiology and shaping the peripheral and central immune/inflammatory responses in CNS diseases [472]. Indeed, there is innovatory evidence supporting the existence of a microbiota-gut-inflammasome-brain axis, in which enteric bacteria modulate, *via* NLRP3 signaling, inflammatory pathways that, in turn, contribute to influence brain homeostasis and neurodegenerative diseases like PD [473]. Considering this, it is interesting to note that meth activates the NLRP3 inflammasome and promotes the processing and release of interleukin (IL)-1β, resulting in neurotoxic activity [474]. Of note is the additional fact that all the intestinal inflammatory changes due to meth depend on the overexpression of NLRP3 inflammasome, causing severe intestinal inflammatory injury *via* NLRP3 inflammasome overexpression [475].

Using high-throughput RNA sequencing in intestinal samples from meth-treated mice, key molecules that might be involved in the pathogenesis of a special type of meth-induced inflammatory bowel disease (IBD) have been identified [476], while a very recent retrospective cohort study analyzed a significant association between IBD and the subsequent development of PD [477].

Overall, it appears that changes in gut microbiota can promote enteric and peripheral neurogenic/inflammatory responses, which, in turn, could contribute to neuroinflammation and neurodegeneration in the CNS. This supports the hypothesis that the pathological process of PD can spread from the gut to the brain. Because meth can induce some alterations in the gut microbiota *via* the aforementioned molecular mechanisms/pathways, one can reasonably propose that meth intoxication may significantly enhance the probability of developing PD pathology *via* the gut-brain axis.

### 4.3 Clinical Evidence Supporting that Meth Could be a Risk Factor for PD

Given the worldwide eminence of illicit meth abuse and the fact that meth predominantly and selectively damages the nigrostriatal pathway when used continually in high doses, it has been speculated that chronic meth/amphetamine users may have an above-normal risk for developing PD and Parkinsonism [88, 478], mostly because meth impairs DA neurons in the substantia nigra similar to the pathological manifestations occurring in PD cases. This section discusses the most recent clinical evidence reinforcing the postulate that meth-induced neurodegenerative changes taking place in human drug abusers are similar to PD pathogenesis.

Meth abuse undoubtedly causes significant long-term dopaminergic neurotoxicity and neurodegeneration in human abusers [479-481], which led to the question of whether there exists a correlation between meth abuse and susceptibility to later develop PD. In this context, the presence of several markers of PD pathogenesis in meth abusers is going in that direction [482].

A population-based cohort study using inpatient hospital discharge records of meth users followed up for about 10 years showed an increased risk of subsequent admission with PD compared to their respective control groups [483, 484]. In the same manner, a retrospective design used to examine meth/amphetamine cohort studies from medical records linked to the Utah Population Database showed a nearly three-fold increased risk of PD in meth/amphetamine users was observed in comparison to population-based controls [485].

In a cross-sectional, observational study, transcranial sonography was used to assess the echogenicity and frequencies of an abnormal spatial extension of the substantia nigra in meth abusers. It was observed that the average echogenic size of the substantia nigra was larger in meth users consorted by increased frequency of echogenic substantia nigra [486]. Additionally, a case study demonstrated an association between meth and Parkinsonism where an MRI analysis of a patient who developed persistent Parkinsonism post-IV inoculations of high-dose meth revealed bilateral hypoxic/ischemic basal ganglia damage [487]. Another case report described chronic meth-induced Parkinsonism as a subacute syndrome that mimics PD affecting the associated neuronal networking [488], while a case study of a 29-year-old female who developed Parkinsonism revealed marked basal ganglia edema and necrosis associated with crystal meth abuse, which clearly indicated that illicit meth use basal ganglia toxicity leads to clinical Parkinsonism [489].

Stigma regarding drug use related to any disorder is counterproductive, and some preliminary research suggests that in low pharmaceutical-grade doses, meth may actually repair and protect the brain in certain pathological circumstances like stroke and traumatic brain injury. In fact, over-the-counter nasal decongestant contains levomethamphetamine and selegiline, a drug for treating PD [490], also metabolizes into levomethamphetamine. To add on, in some cases, it has been reported that meth induces neurogenesis in neuronal subpopulations of the mouse striatum, although such revelations need more investigations regarding the functional capacities of the newly formed neurons [491]. It is in any manner obvious that the dose and route of administration guide the observed outcome of the effects of the drug.

## CONCLUDING REMARKS

**Fig. (3):**
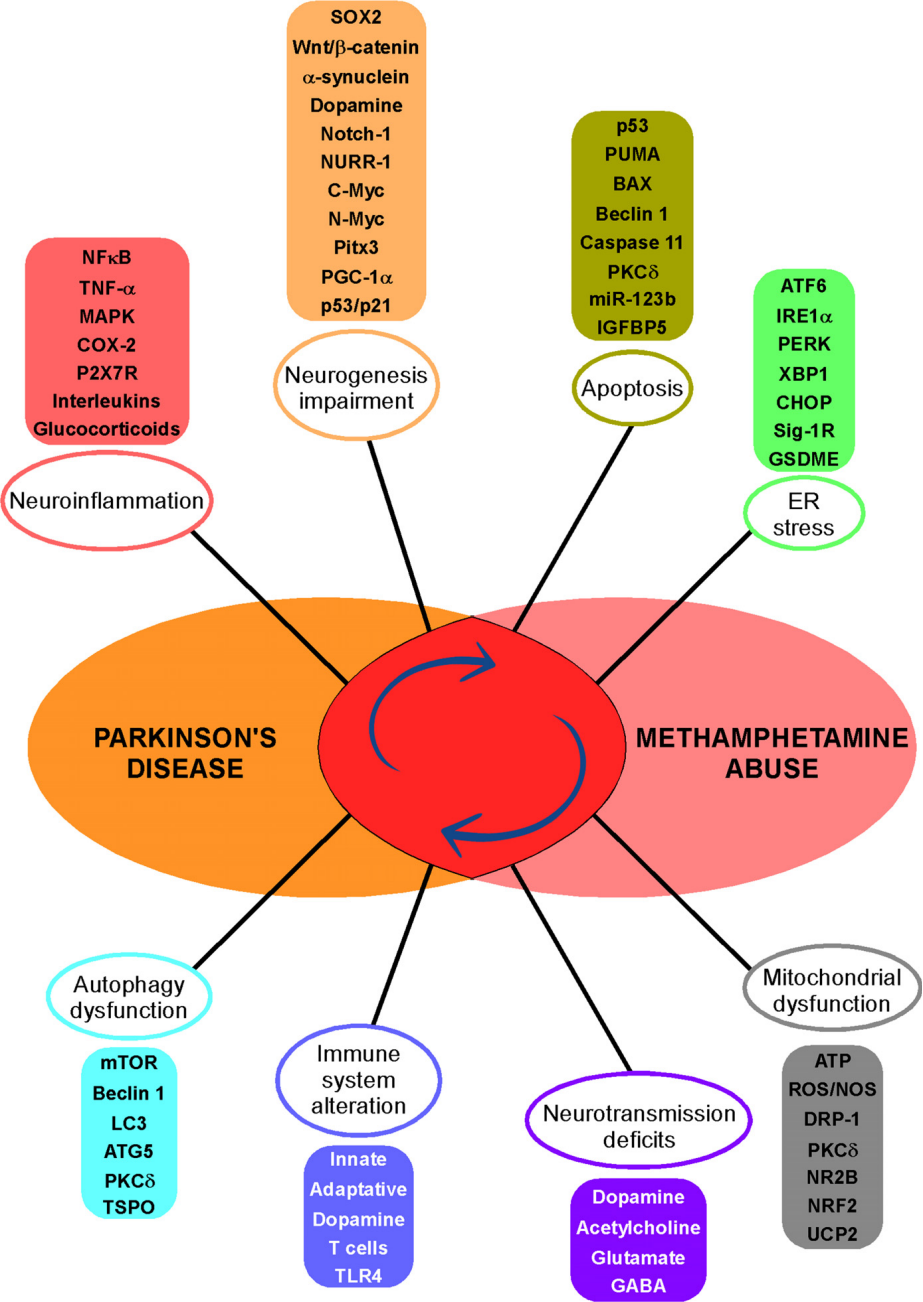
An overview of the prevalent underlying factors similarly implicated in PD pathogenesis and meth abuse. Numerous factors involved in neuroinflammation, neurogenesis, apoptosis, ER stress, autophagy, immunity, neurotransmission and mitochondrial homeostasis, are similarly affected in meth abuse and PD, thereby strongly suggesting that consuming large amounts of this drug in adolescence or adulthood increases the risk of developing PD at later stages of life.

A consistent number of molecular processes linking meth abuse with PD have been established in recent years (Fig. **3**), thereby strongly reinforcing the idea that meth abuse might be a genuine risk for the subsequent development of PD at an older age. However, a substantial amount of data, especially more large-scale clinical studies, is further required to draw definitive conclusions on this subject. Nevertheless, it is of particular interest to note that although the vast majority of the meth-induced effects are neurotoxic and disease-promoting, it remains that there could be a narrow anti-PD therapeutic window of action depending on the signalling cascade considered and the dose of meth applied. This area of research would certainly deserve some particular attention in the future.

## Figures and Tables

**Table 1:** Genetic linkage between PD and meth abuse.

**Factor**	**PD**	**Meth Abuse**	**References**
**SNCA/** **α-synuclein**	*SNCA* polymorphism associated with the risk of developing PD	*SNCA* polymorphism with meth psychosis Meth ↗ by 3x the risk of developing PD by inducing α-synuclein Meth ↗ demethylation of *SNCA* promoter to ↗ α-synuclein levels	[48] [49] [42] [46] [50]
**BDNF**	↗ neuroprotection in PD Val66Met polymorphism is linked to cognitive decline in PD	Meth ↗ BDNF gene methylation to ↘ its expression Val66Met polymorphism is a risk for meth abuse	[123] [124] [52] [51] [55]
**RGS9**	High in PD	Involved in meth-induced psychosis	[56] [57]
**GBA1**	GBA1 heterozygous mutations are linked to PD development Epigenetic modifications and genetic variations in GBA1 are involved in DLB	Expression is ↘ by meth	[60] [59] [61]

**Table 2:** Biomarkers and genetic/epigenetic common denominators to PD and meth abuse.

**Factor**	**PD**	**Meth abuse**	**References**
**Parkin**	Loss of function and/or mutations in parkin are associated with autosomal recessive forms of PD	Meth ↘ the expression of parkin	[111] [112]
**UCHL1**	PD-associated gene	Meth ↗ UCHL1 cortical expression	[121] [120]
**HATp300**	Enhances the aggregation of misfolded proteins in PD	Meth ↗ HATp300 expression in the nucleus accumbens	[122] [103]
**miR-let-7e**	Increasing the level of let-7 attenuates the pathogenic effects of LRRK2	Meth ↘ the levels of miR-let-7e in the plasma	[117] [118]
**miR-128/** **EEATs**	Dysfunction of EAATs and alterations in their expression in PD animal models miR-128 expression affects apoptotic mechanisms in DA neurons along with the expression of excitatory amino acid transporter 4 (EAAT4)	miR-128 is involved in regulating meth sensitization through the control of neuroplasticity	[136] [132] [134]
**miR-181a**	miR-181a suppresses parkin- mediated mitophagy	Meth ↘ miR-181a expression miR-181a is indirectly responsible for meth addiction through the regulation of ER-associated protein degradation	[135] [118] [137]
**miR-124**	miR-124 is decreased in mouse model of PD	miR-124 is associated with meth addiction	[138] [139]
**miR-212-3p**	miR-212-3p is down regulated in PD	miR-212-3p is decreased in the nucleus accumbens of meth- treated mice	[140] [119]
**TET**	Epigenetic and transcriptional ↗ of TET2 in PD patients	Meth ↗ hydroxymethylation of certain genes in nucleus accumbens in a TET1- and TET3-dependent manner	[106] [107]
**HDAC2**	↗ of HDAC2 in the substantia nigra of PD patients	Meth ↗ HDAC2 protein levels in the nucleus accumbens	[108] [103]
**Argonaute 2**	Reduced argonaute 2 levels in the brain of PD patients	Meth ↘ argonaute 2 mRNA and protein levels	[129] [127]
**H3K4me3**	H3K4me3 is ↗ at the SNCA promoter of the substantia nigra of PD patients	Meth ↗ the expression of genes with promoters marked by H3K4me3	[113] [114]

**Table 3:** Similarities regarding endoplasmic reticulum and mitochondrial stress in PD and meth abuse.

**Factor**	**PD**	**Meth Abuse**	**References**
***Endoplasmic reticulum stress***	Signs of ER stress observed in post-mortem tissue from sporadic human PD cases and in most animal models of the disease PD is intimately linked to ER stress and unfolded protein response (UPR) activation	Positive correlation between ER stress and meth-induced neurotoxicity Meth exposure leads to ER stress in dopaminergic cells	[162] [161] [166] [167]
**ATF6**	ATF6 has a functional role in controlling dopaminergic neuron survival in neurotoxin-based models of PD *in vivo*	Meth is able to mediate ER stress *via* the ATF6 signaling pathway	[163] [165]
**Sig-1R**	Sig-1R has been considered as a potential target for PD as it regulates mechanisms of both cellular defense and damage	Sig-1R is involved in meth-induced microglial apoptosis	[35] [168]
**α-synuclein**	α-synuclein oligomers exert neurotoxicity and promote neurodegeneration *via* ER stress and proteostasis dysregulation	Meth persistently increases α-synuclein	[164] [50]
**CHOP/XBP1**	Both have a functional role in controlling dopaminergic neuron survival in neurotoxin-based models of PD *in vivo*	Meth ↗ ER stress through the overexpression of CHOP and spliced XBP1 Up regulation of CHOP in long-term meth abusers	[163] [169] [171]
**GSDME**	Role of GSDME in converting non-inflammatory apoptosis to pyroptosis Pyroptosis is detectable in the biological fluids of PD patients	Meth induces GSDME-dependent ER stress in hippocampal neuronal cells	[175] [174] [176]
**Blood brain Barrier (BBB)**	ER stress-mediated BBB damage occurs in PD	Meth causes damage to BBB *via* ER stress	[145] [177]
***Mitochondrial stress***	Mitochondrial dysfunction is a key determinant of dopaminergic neuronal susceptibility in familial and sporadic PD Mitochondrial stress is associated with α-synuclein pathogenicity	Meth ↗ free radical burden and ↘ neuronal energy supplies Meth ↗ mitochondrial fragmentation Meth ↘ mitochondrial cytochrome c and mitochondrial membrane potential Meth disrupts mitochondrial functions Meth induces PKCδ-dependent mitochondrial dysfunctions Meth induces NFκB-dependent mitochondrial dysfunctions Meth induces LCN2-mediated mitochondrial-related neuronal apoptosis Meth causes a dysfunction in the respiratory chain of the mitochondria in an UCP2-dependent manner Meth ↗ (DRP1)-dependent mitochondrial fragmentation	[189] [190] [199] [192] [193] [205] [201] [194] [202] [206] [210] [211]
**Parkin**	Parkin can rescue mitochondrial pathology due to PINK1 loss of function PD-associated parkin loss of function leads to mitochondrial dysfunction	Meth ↘ parkin levels	[185] [186] [188]
**NMDA/NR2B**	Degeneration of the dopaminergic nigrostriatal pathway in PD ↗ transmission of NR2B-containing NMDA receptors Glutamatergic hyperactivity was observed in the nigrostriatal pathways in PD	Meth ↗ NR2B expression and the level of glutamate in the ventral tegmental area (VTA) and nucleus accumbens (NAc)	[196] [198] [197]
**DRP1**	Reduced functioning of DRP1 in transgenic mouse model of PD is associated with α-synuclein pathology	Meth-induced ↘ of parkin ↘ DRP1 levels	[187] [188]
**Nrf2**	Beneficial effects of Nrf2 expression in inhibiting the progression of PD in 6-OHDA exposed rats	Meth ↘ the activity of Nrf2 and the expression of its downstream proteins	[204] [188]
**LCN2**	LCN2 protein levels are ↗ in the substantia nigra and in the serum of PD patients	Meth ↗ LCN2 in hippocampal astrocytes, CSF and serum in rats	[207] [208] [206]

**Table 4:** Factors involved in immune system functions, neuroinflammation and autophagy that are modified in PD and meth abuse.

**Factor**	**PD**	**Meth Abuse**	**References**
***Immune system***	α-synuclein is involved in the activation of innate and adaptive immunity	Meth impacts the host immune system	[218] [220]
***Neuroinflammation***	Chronic neuroinflammation is one of the hallmarks of PD	Meth activates neuroinflammatory cascades in the brain *Inflammatory profiles in brain and gut are similar in PD and meth abuse*	[226] [232] [249]
**TNFα**	TNFα is ↗ in PD	Meth ↗ TNFα expression and release	[229] [233] [234] [248]
**IL6**	IL-6 is ↗ in the nigrostriatal region and in the CSF of PD patients	IL-6 is ↗ in individuals with meth- associated psychosis and correlates with the severity of cognitive functions	[235] [236]
**COX-2**	COX-2 is associated with PD	Meth ↗ COX-2	[240] [239]
**P2X7R**	P2X7R signaling mediates dopaminergic cell death	Meth stimulates μglial activation through P2X7R	[242] [244]
**HPA axis**	Imbalanced HPA in PD patients	Meth alters HPA axis	[245] [246]
**Peli1/** **NFκB/MAPK**	↗ in the substantia nigra of the human and mouse PD brains	Meth ↗ NFκB and MAPK pathways and pro-inflammatory cytokines	[273] [274]
**PKCδ**	PKCδ KO ↘ neuroinflammation in PD mouse model	Meth ↗ PKCδ expression and activity	[271] [270] [303]
***Autophagy***	Association between PD and autophagy-related genes Autophagy has been associated with PD pathogenesis	Relationship between meth toxicity and mechanisms associated with autophagy Meth influences apoptotic autophagy of dopaminergic neurons	[252] [251] [257] [258]
**α-synuclein**	Autophagy dysfunction is associated with the aggregation of α-synuclein in PD brain	Meth can transfer pathological α-synuclein from neurons to astrocytes *via* exosomes	[253] [254]
**Chaperone-mediated autophagy**	Its activity declines in PD	It is reduced by meth in neurons	[255] [256]
**LC3**	LC3 participates to autophagosome build up and lewy bodies formation in the substantia nigra of PD brains	Meth ↘ the activity of Nrf2 and the expression of its downstream proteins	[263] [261]
**mTOR**	Dysregulation of mTOR in PD pathogenesis	Meth inactivates the mTOR pathway	[262] [260]

**Table 5:** Factors involved in apoptosis that are similarly modified in PD and meth abuse.

**Factor**	**PD**	**Meth Abuse**	**References**
***Apoptosis***	Apoptosis is a key event in PD pathogenesis	Meth induces apoptosis	[278] [279] [287]
**PINK1**	Mutations in PINK1 are linked to early onset Parkinsonism	Meth-induced apoptosis is reversed by functional but not by non-functional PINK1	[286] [288]
**p53**	Depletion or PD-associated mutations of parkin ↗ p53 p53 is involved in PD etiology	p53 is involved in long-term deleterious effects of meth on DA system Meth ↗ p53	[282] [280] [281] [291] [297]
**PUMA**	Involved in the apoptotic mechanisms taking place in PD	Meth ↗ PUMA	[283] [284] [291]
**miR-133b**	↘ of the anti-apoptotic miR-133b in PD	Meth ↘ miR-133b	[293] [294]
**c/EBPβ**	c/EBPβ/δ mediates PD pathogenesis	Meth ↗ C/EBPβ	[296] [295]
**α-synuclein**	Phosphorylation of α-synuclein at serine 129 is one hallmark of PD	Meth ↗ α-synuclein phosphorylation at serine 129 and ↗ α-synuclein aggregation and apoptosis	[298] [299]
**PKCδ**	Caspase-3-dependent activation of PKCδ ↗ apoptosis in DA neuronal cells Suppression of caspase-3-dependent activation of PKCδ ↘ DA neurons degeneration PKCδ inhibition ↘ neuronal loss in the MPTP mouse model of PD PKCδ is ↗ in the brain of PD patients PKCδ knockout ↘ nigrostriatal dopamine degeneration in the MPTP mouse model of PD	Meth ↗ PKCδ PKCδ inhibition ↘ meth-induced apoptosis in mice Meth ↗ mitochondrial translocation of PKCδ and apoptosis in mice	[300] [301] [272] [271] [271] [269] [270] [303] [270] [302]
**Caspase-11**	Mediates dopaminergic cell death in a PD mouse model	Plays essential roles in meth-induced dopaminergic neuronal apoptosis	[289] [290]

**Table 6:** Neurotransmitters pathways similarly modified in PD and meth abuse.

**Factor**	**PD**	**Meth Abuse**	**References**
***Cholinergic transmission***	Cholinergic dysfunctions in PD	Meth impairs cholinergic transmission	[316] [317] [319] [320] [321] [322] [323] [324] [325] [326]
**α7β2 nAChR**	The partial α7β2 nAChR agonist varenicline improves attention in PD patients	Varenicline ameliorates choice strategy and decision making in meth-treated rats	[327] [328]
***Dopaminergic transmission***	Dopaminergic alteration in PD	Meth induces long-term damage to nigrostriatal dopaminergic neurons Meth triggers changes in the mesolimbic dopaminergic system Meth perturbs dopaminergic transmission by blocking DA reuptake Meth alters hippocampal functions to alter memory and learning Meth induces dopaminergic damage *via* a D1 receptor-mediated activation of autophagy	[306] [307] [308] [310] [31] [311]
**Calcium signaling**	Dopaminergic neurons display large cytosolic Ca^2+^ oscillations in PD	Meth regulates Ca^2+^-activated potassium channel activity	[313] [312]
***Glutamatergic/ GABAergic transmission***	Glutamate/GABA imbalance of in PD	Meth alters the levels of glutamate/ glutamine and GABA in the prefrontal cortex Meth alters NMDA and AMPA glutamate receptors in the hippocampus, striatum and frontal cortex Meth elicits an increase of endogenous glutamate in the brain	[335] [336] [337] [343] [344] [345] [342] [358] [359] [341]
**GLT-1**	GLT-1 deficiency in PD	Psychostimulants (including meth) ↘ GLT-1	[351] [352]
**mGluR5**	mGluR5-calcium-dependent cascade causes axonal degeneration in PD	mGluR5 receptors mediate meth- dependent drug-seeking behavior	[353] [354] [355]

**Table 7:** Factors involved in adult neurogenesis that are similarly modified in PD and meth abuse.

**Factor**	**PD**	**Meth Abuse**	**References**
***Neurogenesis***	Affected in PD	Affected by meth Abstinence from meth addiction increases adult neurogenesis in the dentate gyrus	[389] [407] [408]
**NURR1**	NURR1 ↗ the differentiation of NSCs into DA neurons Loss of function NURR1 mutations in PD patients	Meth ↘ NURR1 expression Reduced NURR1 levels exacerbate meth-induced acute and long-term toxicity	[410] [411] [409] [412] [254] [414]
**C-Myc**	C-Myc is ↗ in reactive astrocytes of the substantia nigra of PD patients	Meth ↗ C-Myc	[415] [416]
**N-Myc**	C-Myc is ↗ in reactive astrocytes of the substantia nigra of PD patients Parkin expression has an inverse relation with N-myc levels	N-Myc is associated with meth- induced neuronal injury	[415] [417] [418]
**Wnt/β-catenin**	Overexpression of the Wnt signaling pathway inhibitor DKK1 in PD Wnt/β-catenin is required for the neurogenesis of DA neurons Wnt/β-catenin degenerates in PD	Meth ↘ Wnt/β-catenin signaling Meth ↗ the expression of DKK1	[433] [428] [429] [431] [432]
**Nrf2**	Nrf2 is positively controlled by miR-7 that is highly expressed in TH-positive DA neurons	Meth ↘ Nrf2	[441] [352]
**p53/p21**	PD-associated LRKK2 mutation G2019S ↗ p53/p21 and cellular senescence	Suppression of caspase-3-dependent activation of PKCδ	[445] [444]
**Bax**	Bax ablation ↘ dopaminergic neurodegeneration in a PD mouse model Deletion of Bax ↗ adult neurogenesis and ↘ PD-asssociated anxiety-like behaviors	Meth ↗ Bax Meth ↗ anxiety-like behaviors	[437] [438] [440] [439]

## LIST OF ABBREVIATIONS

AADC: Aromatic l-amino Acid Decarboxylase
AD: Alzheimer’s Disease
Akt: Protein Kinase B
AMPA: α-Amino-3-hydroxy-5-methyl-4-isoxazole propionic acid
AMPK: AMP-activated Protein Kinase
AP1: Activator Protein 1
APOE: Apolipoprotein E
ATF6: Activating Transcription Factor 6
Atg: Autophagy-related Protein
ATP: Adenosine Triphosphate
Aβ: Amyloid Beta
*BAX*: Bcl-2-like Protein 4
BBB: Blood Brain Barrier
*Bcl-2*: B-cell Lymphoma-2
BDNF: Brain-derived Neurotrophic Factor
BIS: Barratt Impulsiveness Scale
C/EBPβ: CCAAT-enhancer Binding Protein Beta
CA: Cornu Ammonis
CAMKII: Ca^2+^/calmodulin-dependent Protein Kinase II
CD36: Cluster of Differentiation 36
CDK5: Cyclin-dependent Kinase 5
CHOP: C/EBP Homologous Protein
CNS: Central Nervous System
COX: Cyclooxygenase
CSF: Cerebrospinal Fluid
CYP2D6: Cytochrome P450 2D6
DA: Dopamine
DAT: DA transporter
DJ1: Protein Deglycase *DJ-1*
*DKK1*: Dickkopf-related Protein 1
DLB: Dementia With Lewy Bodies
DNA: Desoxyribonucleic Acid
DRD2: Dopamine Receptor D2
DRP1: Dynamin-related Protein 1
EAAT4: Excitatory Amino Acid Transporter 4
ENS: Enteric Nervous System
EpCAm: Epithelial Cell Adhesion Molecule
ER: Endoplasmic Reticulum
*ERK*: Extracellular Signal-regulated Kinase
FABP1: Fatty Acid-Binding Protein *1*
*fMRI*: Functional *Magnetic Resonance Imaging*
*FOXO*: Forkhead Box Protein
*GABA*: γ-Aminobutyric Acid
GABBR1: Gamma-aminobutyric Acid Type B Receptor Subunit 1
GBA1: Glucocerebrosidase
GLT-1: Glutamate Transporter-1
GRIA2: Glutamate Ionotropic Receptor AMPA Type Subunit 2
GSDME: Gasdermin E
GSK: Glycogen Synthase Kinase
H3K4me3: Addition of Three Methyl Groups to the Lysine 4 on the Histone H3 Protein
HAT: Histone Acetyltransferase
HD: Huntington's Disease
HDAC: Histone Deacetylase
HIV: Human Immunodeficiency Virus
HPA: Hypothalamic-pituitary-adrenal Axis
IBD: Inflammatory Bowel Disease
ICAM-1: Intercellular Adhesion Molecule-1
IGFBP: Insulin-like Growth Factor Binding Protein
IL: Interleukin
IRE1α: Inositol-requiring Enzyme-1α
KEGG: Kyoto Encyclopaedia of Genes and Genomes
LC3: Microtubule-associated Protein 1 Light Chain 3
LCN2: Lipocalin 2
LRRK2: Leucine-rich Repeat Kinase 2
LTP: Long Term Potentiation
mAChRs: Muscarinic Acetylcholine Receptors
MAO: Monoamine Oxidase
MAPK: Mitogen-activated Protein Kinase
MAPT: Microtubule Associated Protein Tau
MCI: Mild Cognitive Impairment
MD2: Myelin Differentiation Factor 2
*MDMA*: 3,4-Methylenedioxymethamphetamine
Meth: Methamphetamine
mGluR: Metabotropic Glutamate Receptor
miRNA: Micro Ribonucleic Acid
MLC: Myosin Light Chain
MMP-9: Matrix Metalloproteinase-9
MPP^+^: 1-Methyl-4-phenylpyridinium
MPTP: 1-Methyl-4-phenyl-1,2,3,6-tetrahydropyri-dine
MRI: Magnetic Resonance Imaging
mTOR: Mammalian Target of Rapamycin
NAc: Nucleus Accumbens
nAChRs: Nicotinic Acetylcholine Receptors
NADPH: Nicotinamide Adenine Dinucleotide Phosphate Hydrogen
NeuN: Neuronal Nuclear Protein
NF-κB: Nuclear Factor Kappa Light Chain Enhancer of Activated B Cells
NLRP3: Nucleotide-binding Oligomerization Domain Leucine Rich Repeat and Pyrin Domain-containing Protein 3
NMDA: N-methyl-D-aspartate
NOX2: NADPH Oxidase 2
NPCs: Neural Progenitor Cells
NR2B: NMDA Receptor Subunit 2B
Nrf2: Nuclear Factor Erythroid 2-related Factor 2
NSCs: Neural Stem Cells
NUPR1: Nuclear Protein 1
NURR1: Nuclear Receptor Related 1
Oct-4: Octamer-binding Transcription Factor-4
6-OHDA: 6-hydroxydopamine
P2X7R: Ionotropic Purinoceptor 7
PAKs: p21-activated Kinases
PARK: Parkinson Disease Protein
PC12: Pheochromocytoma 12
PD: Parkinson's Disease
PDD: Parkinson’s Disease Dementia
PERK: Protein Kinase RNA-like Endoplasmic Reticulum Kinase
PET: Positron Emission Tomography
PFC: Prefrontal Cortex
PGC-1α: Peroxisome Proliferator-activated Receptor-γ Coactivator-1 Alpha
PHOX: Phosphate-repressible Alkaline Phosphatase
PINK1: Phosphatase and TENsin Homolog-induced Kinase 1
Pitx3: Pituitary Homeobox 3
PKC: Protein Kinase C
PLC: Phospholipase C
PNS: Peripheral Nervous System
POLG: DNA Polymerase Subunit Gamma
PP2A: Protein Phosphatase 2A
PPP: Pentose-phosphate Pathway
PUMA: p53 Upregulates Modulator of Apoptosis
RGS9: Regulator of G-protein Signalling 2
RNA: Ribonucleic Acid
ROCK1: Rho-associated Protein Kinase 1
ROS: Reactive Oxygen Species
S100B: Calcium-binding Protein B
SCF: Skp1-Cul1-F box Protein
SCFAs: Short-chain Fatty Acids
scRNA-seq: Single-cell RNA Sequencing
Sig-1R: Sigma-1 Receptor
SNCA: Synuclein Alpha
SNpc: Substantia Nigra Pars Compacta
*SOX2*: (SRY-sex Determining region Y)-box Transcription Factor 2
SVZ: Subventricular Zone
TBK1: TANK-binding Kinase 1
TET: Ten-eleven Translocation
TH: Tyrosine Hydroxylase
TLR: Toll-like Receptor
TNFα: Tumour Necrosis Factor Alpha
TOM: Translocase of the Outer Membrane
*TREM*: Triggering Receptor Expressed on Myeloid Cells
Trib3: Tribbles Homolog 3
TRIF: TIR-domain-containing Adapter-inducing Interferon-β
TSPO: 18 kDa Translocator Protein
UCHL1: Carboxy-terminal Hydrolase L1
UCP2: Uncoupling Protein 2
*ULK1*: Unc-51-like Autophagy Activating Kinase 1
UPR: Unfolded Protein Response
UPS: Ubiquitin-proteasome System
VCAM-1: Vascular Cell Adhesion Molecule-1
VMAT2: Vesicular Monoamine Transporter Type 2
VTA: Ventral Tegmental Area
XBP1: X-box Binding Protein 1
